# Differential Subcellular Localization of *Leishmania* Alba-Domain Proteins throughout the Parasite Development

**DOI:** 10.1371/journal.pone.0137243

**Published:** 2015-09-03

**Authors:** Aurélien Dupé, Carole Dumas, Barbara Papadopoulou

**Affiliations:** Research Center in Infectious Diseases, CHU de Quebec Research Center-Université Laval and Department of Microbiology, Infectious Diseases and Immunology, Laval University, Quebec, QC, Canada; Instituto Oswaldo Cruz, Fiocruz, BRAZIL

## Abstract

Alba-domain proteins are RNA-binding proteins found in *archaea* and eukaryotes and recently studied in protozoan parasites where they play a role in the regulation of virulence factors and stage-specific proteins. This work describes in silico structural characterization, cellular localization and biochemical analyses of Alba-domain proteins in *Leishmania infantum*. We show that in contrast to other protozoa, *Leishmania* have two Alba-domain proteins, *Li*Alba1 and *Li*Alba3, representative of the Rpp20- and the Rpp25-like eukaryotic subfamilies, respectively, which share several sequence and structural similarities but also important differences with orthologs in other protozoa, especially in sequences targeted for post-translational modifications. *Li*Alba1 and *Li*Alba3 proteins form a complex interacting with other RNA-binding proteins, ribosomal subunits, and translation factors as supported by co-immunoprecipitation and sucrose gradient sedimentation analysis. A higher co-sedimentation of Alba proteins with ribosomal subunits was seen upon conditions of decreased translation, suggesting a role of these proteins in translational repression. The *Leishmania* Alba-domain proteins display differential cellular localization throughout the parasite development. In the insect promastigote stage, Alba proteins co-localize predominantly to the cytoplasm but they translocate to the nucleolus and the flagellum upon amastigote differentiation in the mammalian host and are found back to the cytoplasm once amastigote differentiation is completed. Heat-shock, a major signal of amastigote differentiation, triggers Alba translocation to the nucleolus and the flagellum. Purification of the *Leishmania* flagellum confirmed *Li*Alba3 enrichment in this organelle during amastigote differentiation. Moreover, partial characterization of the *Leishmania* flagellum proteome of promastigotes and differentiating amastigotes revealed the presence of other RNA-binding proteins, as well as differences in the flagellum composition between these two parasite lifestages. Shuttling of Alba-domain proteins between the cytoplasm and the nucleolus or the flagellum throughout the parasite life cycle suggests that these RNA-binding proteins participate in several distinct regulatory pathways controlling developmental gene expression in *Leishmania*.

## Introduction

Trypanosomatids are kinetoplastid protists that are responsible for a variety of diseases affecting tropical and subtropical regions around the world. These include sleeping sickness caused by *Trypanosoma brucei*, ‘Chagas’ disease caused by *Trypanosoma cruzi*, and different forms of leishmaniasis caused by *Leishmania* spp. *Leishmania infantum* is one of the pathogenic agents of visceral leishmaniasis, the most severe form of the disease, which is responsible for more than 500 000 new cases and approximately 50 000 deaths annually [1]. *Leishmania* metacyclic promastigotes are injected into the skin of the mammalian host by the bite of sandflies and internalized in the phagolysosome of macrophages where they differentiate into amastigotes that are adapted to replicate within the phagolysosomal vacuoles [2, 3]. Changes in temperature between the poikilothermic insect vector and the thermostable mammalian host, and the acidic environment of the phagolysosomal compartment trigger amastigote differentiation [4]. These environmental factors together with differences in nutriment availability in the phagolysosome are responsible for differential regulation and expression of many sets of genes [5, 6].

The genome organization of *Leishmania*, trypanosomes, and related kinetoplastid organisms is unusual. *Leishmania* genes are organized as large directional polycistronic clusters comprising up to hundreds genes of non-related functions [7]. Individual mRNAs are resolved by two coupled RNA-processing reactions: *trans*-splicing of a capped spliced-leader (SL) at the 5'-end and polyadenylation at the 3'-end (reviewed in [8]). Control does not take place at the level of transcription initiation and most genes are regulated post-transcriptionally by c*is*-acting elements located in the 3'-untranslated regions (3'UTRs) that determine either mRNA abundance and stability or translational efficiency [9]. Trypanosomatids have a large number of predicted RNA-binding proteins, many of which have no orthologs in other eukaryotes. In trypanosomes (reviewed by [10, 11]) and the related *Leishmania* species [12–16], few RNA-binding proteins (RBPs) have been identified so far to bind specific sets of mRNAs. RBPs seem to play important roles in the parasite development, and in the case of *T*. *brucei*, inducible overexpression of RBP6 resulted in the progression of procyclics to epimastigotes and, within a week of RBP6 expression, to metacyclic trypomastigotes in tsetse fly in the absence of any other developmental triggers [17]. More recently, we have identified Alba-domain proteins that bind to and regulate amastigote-specific transcripts in *Leishmania* [18].

Alba-domain proteins (for Acetylation Lowers Binding Affinity) are found in *Archaea* and eukaryotes and display an unusual evolution history. In *Archaea*, they are nucleosome constituents (i.e. *Sulfolobus acidocaldarius*) and RNA-binding proteins (i.e. *Sulfolobus shibatae* [19]) and are globally defined as small basic proteins. This family was latter phylogenetically subdivided into three groups that include *Archaea* proteins and eukaryotic proteins sharing sequence homologies to RNase P/MRP subunits in higher eukaryotes, Rpp20/Pop7 and Rpp25/Pop6, mainly implicated in tRNA and rRNA maturation. A global inter-species alignment published previously highlights the two features of Alba-domain proteins in DNA and/or RNA binding [20]. More recently, Alba-domain proteins have been characterized in protist parasites where they play a role in the regulation of expression of various virulence factors or stage-specific membrane proteins. In *Plasmodium falciparum*, the RGG-box-containing *Pf*Alba3 binds to DNA in telomeric and subtelomeric regions in an acetylation-dependent manner and modulates the expression of the virulent *var* genes. Interestingly, the four *Pf*Alba proteins re-localize from the cytoplasm to the nucleus in the trophozoite and schizonte stages where they bind to the Telomere-Associated Repetitive Elements (TARE1 and TARE6), also associated to the regulation of *var* genes [21, 22]. In the closely related species *Plasmodium berghei*, Alba proteins have an RNA-binding activity and *Pb*Alba1-3 are part of RNP complexes of translationally silent transcripts in oocyte P-granules [23]. In *T*. *cruzi*, the association of some Alba-domain proteins with polysomal-enriched fractions suggests their interaction with the translation machinery [24]. In *T*. *brucei*, the four *Tb*Alba proteins interact with the procyclic-specific *GPEET* transcript and seem to play a role in its translation inhibition [25]. Interestingly, the RGG-box-containing *Tb*Alba proteins are present in starvation and heat-stress granules and seem to play a more global role in cell organization, involving the aborted nucleus migration during the *T*. *brucei* differentiation [26]. Our previous work in *Leishmania infantum* showed that the RGG box-containing *Li*Alba3 (LinJ.34.2410, ortholog of *Tb*Alba3 and *Tb*Alba4) interacts with the 3’UTR of the amastigote-specific *delta-amastin* mRNA [18]. This interaction mediates stabilization of the *amastin* mRNA, specifically in the amastigote stage. On the other hand, *Li*Alba1 (LinJ.13.0270, ortholog of *Tb*Alba1 and *Tb*Alba2) seems to interact with the same mRNA, but depletion of this particular protein does not affect *amastin* mRNA stability [18].

This study describes *in silico* structural characterization, cellular localization and biochemical analyses of Alba-domain proteins in the parasitic protozoan *Leishmania infantum*. The *Leishmania* genome encodes two Alba-domain proteins, *Li*Alba1 (13 kDa) and *Li*Alba3 (20 kDa), instead of four in other protozoan parasites, that physically interact together and associate also with other RNA-binding proteins and components of the ribosome. Alba-domain proteins localize predominantly in the cytoplasm of *Leishmania* promastigote and amastigote life stages, but upon amastigote differentiation they preferentially translocate to the nucleolus and the flagellum, suggesting diverse roles of these RNA-binding proteins in regulating gene expression during the parasite development. Here, we also provide data on the first characterization of the proteome of the flagellar fraction of *Leishmania* promastigotes and differentiating amastigote forms.

## Materials and Methods

### Cell lines and culture

*Leishmania donovani infantum* MHOM/MA/67/ITMAP-263 promastigotes were cultured at 25°C in SDM-79 medium (pH 7.0) supplemented with 10% heat-inactivated fetal calf serum (Multicell, Wisent Inc.) and 5 mg/ml hemin. Axenic amastigotes were grown in MAA/20 medium (pH 5.8) at 37°C with 5% CO_2_ as described [27] and used after two to three passages, when fully differentiated. Differentiating amastigotes were grown for 8 to 24 hours in MAA/20 medium. For transfectant selection, 30 μg/ml G418 and 160 μg/ml hygromycin B were used. For growth phenotype analysis, 10^6^ stationary-phase promastigotes were counted on a Malassez (hemocytometer) and then inoculated in 5 ml of fresh SDM media to measure the O.D._600nm_ every 24 hours. For axenic amastigotes, a similar approach was used but with 10^7^ parasites as a starter. Experiments were done in triplicate, using in average passage 4–5 for promastigotes and passage 3 for amastigotes.

### DNA manipulations and plasmid preparation

*Li*Alba3^-/-^ (LinJ.34.2410; previously named *Li*Alba20^-/-^) and *Li*Alba1^-/-^ (LinJ.13.0270; previously named *Li*Alba13^-/-^) knockout mutants and add-back strains obtained by episomal transfection of plasmids pSP-alphaIRNEOalphaIR-HA-*Li*Alba3 and pSP-alphaIRNEOalphaIR-*Li*Alba1-HA into the corresponding null mutant background were described previously [18]. The strategy used for episomal expression of *Leishmania* proteins of interest fused to fluorescent proteins or for genomic integration is presented in S1A Fig. The fluorescent coding sequence (e.g. mCherry, and yellow fluorescent protein (eYFP)) was fused at the N-terminal part of *Li*Alba1, *Li*Alba3 or *Li*Nop10 to keep the natural context of 3'UTR, hence allowing efficient regulation of the transcripts. Briefly, a first cassette containing a selectable marker gene (*NEO* or *HYG*), the alpha-tubulin intergenic region (alphaIR) for optimal processing and expression of the selectable marker, and the fluorescent protein without the stop codon was prepared by Phusion PCR using Phusion enzyme (Thermo Scientific) according to the manufacturer instructions. Primers (from Integrated DNA Technologies) used for PCR amplifications are listed in S1 Table. The NEO-alphaIR-eYFP and HYG-alphaIR-mCh cassettes were cloned into pGEM-T Easy (Promega) for sequencing and further sub-cloning experiments. The second step consisted in the amplification of a 500 bp fragment corresponding to the 5'-intergenic region of the target gene, and the ORF (cloned in frame with the mCherry and eYFP fluorescent proteins) together with the corresponding 3'-intergenic region (~500 bp). These two fragments were fused by PCR and an EcoRV site was added at the junction. Cloning in pGEM-T Easy and digestion using EcoRV allowed us to perform a blunt insertion of the cassette to finally obtain the pGEM-NEOalphaIR-eYFP-*Li*Alba1, pGEM-HYGalphaIR-mCh-*Li*Alba3 and pGEM-HYGalphaIR-mCh-*Li*Nop10 vectors. The mCh-*Li*Alba3 cassette was amplified from pGEM-HYGalphaIR-mCh-*Li*Alba3 using primers containing XbaI (5'end) and NdeI (3'end) restriction sites. PFR2C-HA was produced by PCR amplification with specific primers harboring the HA sequence and XbaI and NdeI sites. Both constructs (S1B Fig) were sub-cloned into pSP-alphaIRNEOalphaIR XbaI and NdeI sites to generate pSPalphaIRNEOalphaIR-mCh-*Li*Alba3 and pSP-alphaIRNEOalphaIR-PFR2C-HA vectors, respectively as previously described for the Alba-HA constructs [18]. Transfections were performed by electroporation as previously described [28].

### Sequence alignments

All the Alba protein sequences from trypanosomatids were downloaded from TritrypDB.org (v4.0). For both *Li*Alba sequences, protein secondary structure prediction was performed using the Phyre webserver [29]. Alba-domain protein alignments in *Trypanosoma* and *Leishmania* species were carried out with MEGA5 software using ClustalW (Gonnet matrix and 10 gaps allowed). Neighbor-joining tree was build using MEGA6 bootstrap method and Poisson model for substitutions. Phyre results and ClustalW alignment were merged using the BioEdit software.

### Immunofluorescence studies

For indirect immunofluorescence, we used parasites episomally expressing pSP-alphaIRNEOalphaIR-*Li*Alba1-HA and pSP-alphaIRNEOalphaIR-HA-*Li*Alba3 in the *L*. *infantum Li*Alba1^-/-^ and *Li*Alba3^-/-^ null mutant background, respectively. Approximately 10^6^ parasites were centrifuged, washed 3 times with PBS and kept on ice before attachment on microscopy slides for 10 min at room temperature. Cold 100% methanol was used for cell fixation for 10 min at -20°C and 3 x 5 min washes with PBS were performed prior to a 30 min permeabilization at room temperature in the following solution (1X PBS, 0.2% Triton X-100, 5% Fetal Calf Serum). Blocking buffer (1X PBS; 2% BSA; 0.02% Tween-20; 0.1% Triton X-100) was added for overnight incubation at 4°C. An anti-HA antibody (Applied Biological Materials Inc.) diluted 1:400 in blocking buffer was used as primary antibody for 1 hour incubation at room temperature, and unbound material was removed by 5 x 5 min washes in PBS. 2 μg of secondary antibody (Alexa Fluor 488 goat anti-mouse, LiveTech) were used under similar conditions in the dark and washed as described for the anti-HA antibody. For nuclear and kinetoplast DNA staining, DAPI solution (Sigma) diluted to 0.5 μg/ml in 1X PBS was added for 5 min at room temperature and then washed 2 x 5 min with 1X PBS. After drying the slides, a drop of mounting solution (Mobi Glow) and a cover slide were added and sealed with nail polish. For fluorescent protein imaging, we used approximately 10^6^ *L*. *infantum* episomally transfected or co-transfected with pGEM-HYGalpha-mCh-*Li*Alba3, pGEM-NEOalphaIR-eYFP-*Li*Alba1 and/or pGEM-HYGalphaIR-mCh-*Li*Nop10. Parasite cultures were centrifuged, washed in PBS twice, and incubated in 2% paraformaldehyde (Sigma) in PBS for 10 min at room temperature. Cells were dropped on slide and dried prior DAPI staining as described above. After slide drying, Mobi Glow and cover slide were added and sealed with nail polish. All slides were analyzed by epifluorescence microscope using a Nikon Eclipse TE300 with a 100X objective and oil immersion. Images acquisition was performed with ImagePro Plus 5 software, and ImageJ or Photoshop were used for picture merging and montage.

### Co-immunoprecipitation studies

For identifying interacting partners of the Alba-domain proteins, we carried out co-immunoprecipitation studies from recombinant *L*. *infantum* expressing pSP-alphaIRNEOalphaIR-*Li*Alba1-HA or pSP-alphaIRNEOalphaIR-HA-*Li*Alba3 vectors. For amastigotes and promastigotes, 5x10^9^ cells in exponential phase were pelleted and resuspended in 1 ml of cytoplasmic extraction buffer (CEB: 10 mM Hepes, 3 mM MgCl2, 14 mM KCl, pH 7.5, 5% glycerol and 0.2% NP-40), supplemented with 1 mM PMSF and 2X cOmplete ULTRA Tablets Mini, EDTA-free EASYpack (Roche). Cells were lysed with 15 to 20 strokes using a Douncer homogenizer and lysis was verified under the microscope before centrifugation (13 000 rpm) for 10 min at 4°C to remove insoluble material. To avoid identification of proteins bound to the beads, protein extracts were washed on a rotor with 60 μl of Dynabeads Protein G (Invitrogen) for 1 hour at 4°C, and resuspended in 200 μl PSB-0.1% Tween. In parallel, another 60 μl of beads were resuspended in 200 μl PSB-0.1% Tween, incubated with 10 μg of an anti-HA antibody (Applied Biological Materials Inc.) during 1 hour at 4°C on a rotor. Unbound antibody was removed by three washes of 5 min in 500 μl PSB-0.1% Tween and the “washed”proteins were added to the beads and antibody mix. Incubation was carried out for 90 min at 4°C on a rotor and unbound material was removed by 5 washes using PSB-0.1% Tween supplemented with 1X cOmplete ULTRA Tablets Mini, EDTA-free EASYpack. The mix was transferred to a new Eppendorff tube and elution was performed by direct addition of 60 μl of 2x Laemmli Sample Buffer and incubation for 5 min at 100°C. Elutions were loaded on 12% SDS-PAGE and migration was stopped as soon as the bromophenol blue marker entered to the resolving part of the gel. After Coomassie staining and destaining (to remove traces of SDS), bands were excised and conserved in 1% acetic acid for MS/MS peptide identification (see below). The results presented in Table 1 are only peptides corresponding to proteins co-immunoprecipitated with the HA-Alba proteins and are absent from the wild type negative control (data not shown). Immunoprecipitations from *L*. *infantum* wild type, HA-*Li*Alba3 and *Li*Alba1-HA were all run in triplicates.

**Table 1:** *L*. *infantum* Alba-domain protein putative partners identified by co-immunoprecipitation and LC-MS/MS analysis.

TriTrypDB ID	Identified Proteins	Peptide No.^a^ (Probability score 99.9%)			
		*L*. *infantum* promastigotes		*L*. *infantum* amastigotes	
		Alba1-HA	HA-Alba3	Alba1-HA	HA-Alba3
LinJ.34.2410	hypothetical protein, conserved **| *Li*Alba3**	16^b^	15	15	16
LinJ.13.0270	hypothetical protein, conserved **| *Li*Alba1**	11	7	8	6
LinJ.35.5360	polyadenylate-binding protein 1, putative **|PABP1**	4	3	7	3
LinJ.35.4200	poly(A)-binding protein 2,poly(a) binding protein, putative **|PABP2**	12	15	17	12
LinJ.25.0080	poly(A)-binding protein 3 **|PABP3**	3	6	7	7
LinJ.32.0410	ATP-dependent RNA helicase, putative **| Homolog of Dbp1**	3	4	12	10
LinJ.32.0790	RNA binding protein, putative **| NRBD**	4	3	4	6
LinJ.21.0490	hypothetical protein, conserved **|NTF2-like**	2	3	9	9
LinJ.27.1220	hypothetical protein, conserved **| ZC3H41**		2	13	9
LinJ.26.1220	heat shock protein 70-related protein	2	2	9	4
LinJ.36.3010	40S ribosomal protein S24e	4		3	3
LinJ.35.0400	40S ribosomal protein S3A, putative	2		3	2
LinJ.36.5240	40S ribosomal protein SA, putative	4			2
LinJ.28.1050	40S ribosomal protein S14	2		3	3
LinJ.21.2150	40S ribosomal protein S6, putative	3		2	3
LinJ.07.0550	60S ribosomal protein L7a, putative	3	2	2	3
LinJ.29.1160	ribosomal protein L1a, putative			3	3
LinJ.35.2240	RNA-binding protein, putative **| DRBD2**			7	4
LinJ.18.0300	hypothetical protein, conserved **|NTF2-like**			2	
LinJ.25.0550	hypothetical protein SCD6.10 **| Rap-55**			2	1
LinJ.22.1370	40S ribosomal protein L14, putative			3	
LinJ.28.2750	40S ribosomal protein S17, putative			3	3
LinJ.34.2620	40S ribosomal protein S19 protein, putative			2	2
LinJ.15.1010	40S ribosomal protein S3, putative			6	6
LinJ.13.1120	40S ribosomal protein S4, putative			7	4
LinJ.11.0960	40S ribosomal protein S5			3	
LinJ.04.0460	60S ribosomal protein L11 (L5, L16)			3	
LinJ.29.2570	60S ribosomal protein L13, putative			2	3
LinJ.32.4050	60S ribosomal protein L2, putative				4
LinJ.06.0590	60S ribosomal protein L23a, putative			2	2
LinJ.24.2140	60S ribosomal protein L26, putative				3
LinJ.35.1870	60S ribosomal protein L5, putative			3	2
LinJ.30.3390	60S ribosomal protein L9, putative			4	4
LinJ.01.0430	ribosomal protein S7, putative			4	3
LinJ.30.2570	reticulon domain protein, 22 potentially aggravating protein			2	
LinJ.18.0590	RNA binding protein, putative | Leish. specific	2			

^a^Only proteins identified with a minimum of two peptides and a probability of 99.9% to correspond to the correct protein were included here. Identified proteins were grouped into different GO categories based on their predicted function. From those proteins, the ones presented in the upper portion of the Table associate with both *Li*Alba1 and *Li*Alba3 in *L*. *infantum* promastigotes and amastigotes, those in the middle part of the Table associate with *Li*Alba1 and *Li*Alba3 only in amastigotes, and one protein was found to interact with *Li*Alba1 in promastigotes only. The raw data of peptide and spectrum reports are provided in S2 Table.

^b^Each of the three co-IP experiments was run in triplicate. Only proteins identified in two out of the three replicates are presented here, and the number of unique peptides for each protein is representative of three experiments. The maximum number of peptides obtained is indicated here.

### Flagellum purification and flagellar proteome analysis

To purify intact flagella from *Leishmania* parasites, we used the method recently described in [30]. 3x10^9^ parasites were resuspended in 2 mL of Buffer A (25 mM tricine; 0.2 mM EDTA; 5 mM MgCl_2_; 12 mM beta-mercaptoethanol, pH 7.0) supplemented with 0.32 M sucrose, 1% BSA, 0.1 mM CaCl_2_ and protease inhibitor tablet (Roche). To detach the flagellum, cells were vortexed for 10 min at 4°C and centrifuged at 420 g for 10 min. The supernatant was decanted and recentrifuged twice, and the flagella were pelleted on a table top centrifuge at 13,000 rpm for 5 min at 4°C. Sediment was resuspended in 500 μl of Buffer A + 0.32 M Sucrose, layered on top of a discontinuous sucrose gradient of 1.49/1.66/1.84/2.02 M prepared in Buffer A, and centrifuged (28,000 rpm) overnight at 4°C in a Beckman SW40 Ti rotor, and the flagellum were enriched in the 1.66 M sucrose fraction. This fraction was recovered and centrifuged in table top centrifuge at 13,000 rpm for 30 min at 4°C. The pellet was resuspended in Buffer A and centrifuged at maximum speed for 15 min, and finally resuspended in 25 μl of 2X Laemmli buffer and loaded on a 12% SDS-PAGE. Then, the gel was used for Western blot analysis or MS/MS peptide identification as described below.

### Mass spectrometry and database searching

Peptide samples were separated by online reversed-phase (RP) nanoscale capillary liquid chromatography (nanoLC) and analyzed by electrospray mass spectrometry (ES MS/MS). The experiments were performed with Agilent 1200 nanopump connected to a 5600 TripleTOF + mass spectrometer (AB Sciex, Framingham, MA, USA) equipped with a nanoelectrospray ion source. Peptide separation took place on a self-packed PicoFrit column (New Objective, Woburn, MA) packed with Jupiter (Phenomenex) 5u, 300A C18, 15 cm x 0.075 mm internal diameter. Peptides were eluted with a linear gradient from 2–50% solvent B (acetonitrile, 0.1% formic acid) in 30 min, at 300 nL/min. Mass spectra were acquired using a data dependent acquisition mode using Analyst software version 1.6. Each full scan mass spectrum (400 to 1250 m/z) was followed by collision-induced dissociation of the twenty most intense ions. Dynamic exclusion was set for a period of 12 sec and a tolerance of 100 ppm. All MS/MS peak lists (MGF files) were generated using ProteinPilot (AB Sciex, Framingham, MA, USA, Version 4.5) with the paragon algorithm. MGF sample files were then analyzed using Mascot (Matrix Science, London, UK; version 2.4.0) to search the TriTrypDB database (8 May 2014 TriTrypDB 8.0 released). Mascot was searched with a fragment ion mass tolerance of 0.10 Da and a parent ion tolerance of 0.10 Da. Scaffold (version 3.4.5), Proteome Software Inc., Portland, OR) was used to validate MS/MS based peptide and protein identifications. Protein probabilities were assigned by the Protein Prophet algorithm [31]. Proteins that contained similar peptides and could not be differentiated based on MS/MS analysis alone were grouped to satisfy the principles of parsimony.

### Polysome fractionation

Polysome fractionation was analyzed as previously described [32]. Briefly, 3×10^9^ wild type parasites were treated with 100 μg/ml cycloheximide (Sigma) for 10 min and washed with cycloheximide-containing PBS buffer. Cell lysis was performed with a Dounce homogenizer in lysis buffer [10 mM Tris-HCl pH 7.4, 10 mM MgCl2, 150 mM NaCl, 0.5% IGEPAL, 100 μg/ml cycloheximide, 1 mM PMSF, 100 U/ml RNAseOUT (Amersham), 2X final concentration of protease inhibitor tablet (Roche)]. Lysates were divided into two tubes, and one of them was treated 10 min at room temperature with 50 mM EDTA as control. All the lysates were pelleted by centrifugation and the supernatant (40 OD_260 nm_ units) was layered on top of a 15% to 45% linear sucrose gradient (10 ml) prepared in gradient buffer (50 mM Tris-HCl pH 7.4, 10 mM MgCl2, 50 mM KCl, 100 U/ml RNaseOUT). Ultracentrifugation (35000 rpm for 2.15 hours at 4°C) was performed in a Beckman SW40 Ti rotor, and fractions were collected with an ISCO Density Gradient Fractionation System under constant monitoring of the absorbance at 254 nm. Proteins from the various fractions were concentrated using the TCA method, and finally resuspended in 50 μl of 2X Laemmli buffer. 10 μl of each fraction were loaded on 12% SDS-PAGE and transferred for Western blot analysis. Anti-HA (100 ng/ml, Applied Biological Materials Inc.) or anti-*Tb*Alba340/2 (GeneID: Tb927.4.2040 [26],) and HRP-linked anti-mouse (NEB) antibodies were used for 1 h at room temperature.

## Results

### Alba-domain proteins of *Leishmania*

**Fig 1:**
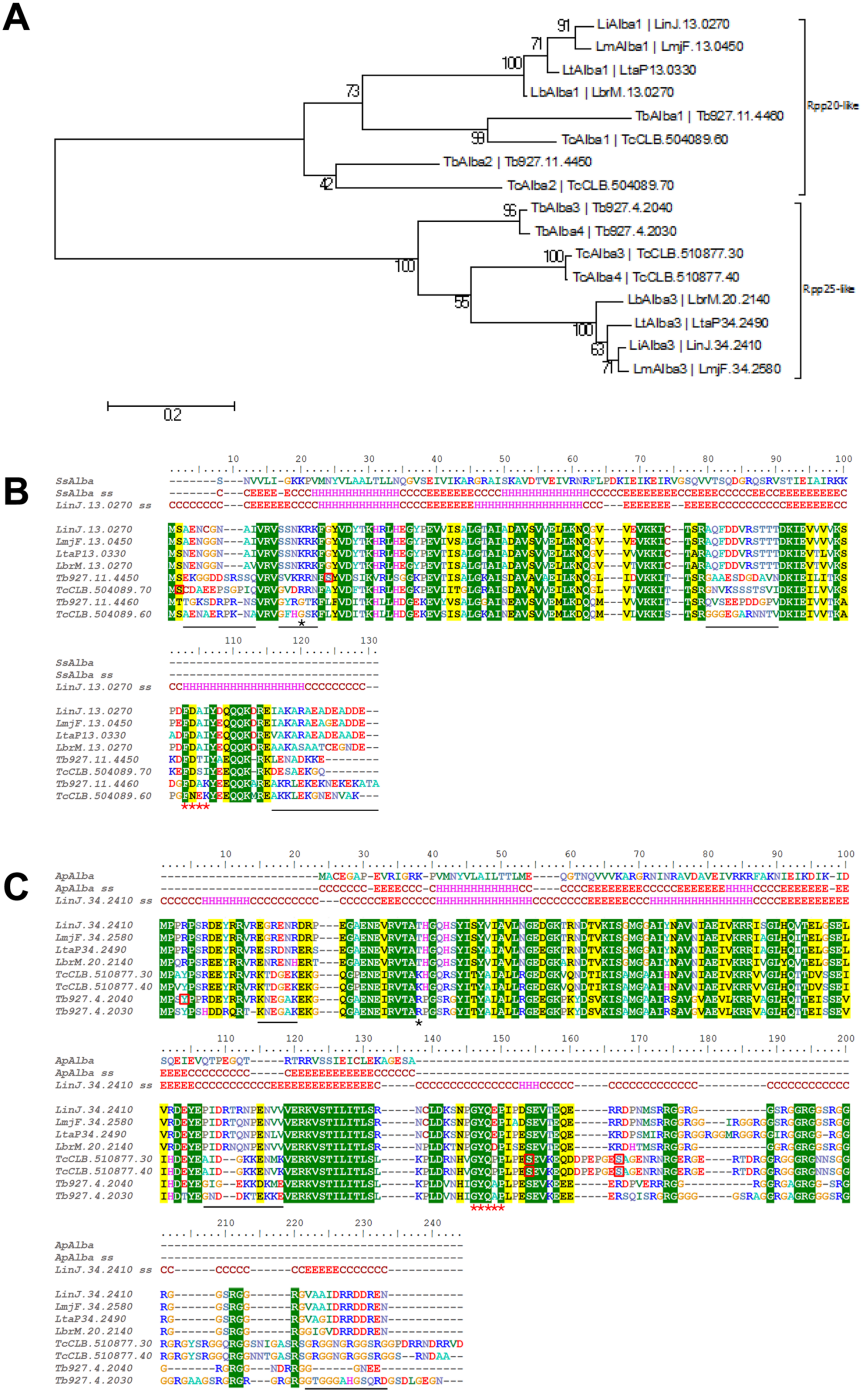
Sequence alignment and phylogeny of Alba-domain proteins in *Leishmania*, *Trypanosoma brucei* and *T*. *cruzi* species. (A) Neighbor-joining tree showing the phylogenetic relationship between the Alba-domain proteins of TriTryps. Evolutional distances (scale) were estimated as the number of amino acid substitutions per site, considering Poisson correction. The two subgroups Rpp20-like and Rpp25-like are marked. (B) ClustalW alignment of Rpp20-like Alba-domain proteins merged with the in silico structure prediction of *Li*Alba1 (LinJ.13.0270) using the Phyre algorithm. The best score was obtained with the Alba protein from *Sulfolobus solfataricus* (*Ss*)(NCBI WP_010923153.1). ss: secondary structure *in silico* prediction; C: coil; H: helix; E: Sheet. Red squares indicate amino acids known to be phosphorylated on these specific genes (*T*. *cruzi* and *T*. *brucei*). The black star shows the expected position for Sir2 acetylation and the red stars underline the signature motif of the subgroup. Sequence variations in coiled regions between *Trypanosoma* spp. and *Leishmania* spp. are underlined with a black bar. *Li*Alba1 was used for Phyre structure prediction. (C) As in B for the Rpp25-like Alba-domain proteins. Structure prediction of *Li*Alba3 using Phyre outputs Alba2 from *Aeropyrum pernix* (*Ap*) K1 (NCBI WP_010866616.1) as the best match. *Li*Alba3 (LinJ.34.2410) was used for Phyre structure prediction. LinJ: *L*. *infantum*; LmjF: *L*. *major*; LtaP: *L*. *tarentolae*; LbrM: *L*. *braziliensis*; Tb: *T*. *brucei*; Tc: *T*. *cruzi*.

In contrast to the *T*. *brucei* genome that encodes four Alba-domain proteins (Alba1-4) corresponding to the Rpp20- and Rpp25-like eukaryotic subgroups, the related *Leishmania* species sequenced to date [7, 33, 34] possess only two Alba-domain proteins (LinJ.13.0270 (*Li*Alba1) and LinJ.34.2410 (*Li*Alba3) in *L*. *infantum*), as illustrated in the Neighbor-joining tree presented in Fig 1A. *Li*Alba1 is part of the Rpp20-like subgroup whereas *Li*Alba3 belongs to the Rpp25-like subgroup. This suggests a duplication of *Alba* genes after *Leishmania* and *Trypanosoma* speciation. In *T*. *cruzi*, similarly to *T*. *brucei*, there are four Alba-domain proteins based on sequence identity (from 65–73%) that are annotated as hypothetical proteins (Fig 1 and not shown).

Members of the Rpp20-like subgroup have a molecular weight of 12–14 kDa. Surprisingly, although they exhibit a basic p*I* in *Trypanosoma* spp., the *Leishmania* orthologs have an acidic p*I* (p*I* 4.8 in the case of *L*. *infantum* compared to 9.8 in *T*. *brucei*). This suggests that *Leishmania* Alba-domain proteins of the Rpp20-like subgroup have different charged amino acids, underlying differences in their abilities to bind their substrates. The charge differences are mainly observed in the C- and N-terminal regions (Fig 1B). Within *Leishmania* species, Rpp20-like proteins are well conserved sharing amino acid sequence identities up to 88–99%. However, identity of *Li*Alba1 protein with *T*, *brucei* Rpp20-like proteins *Tb*Alba2 (Tb11.02.2030) (Tb927.11.4450) and *Tb*Alba1 (Tb11.02.2040) (Tb927.11.4460) drops to 57% and 56%, respectively.

Given the lower primary sequence similarity between the *Leishmania* and *Trypanosoma* Alba proteins and particularly *Li*Alba1 and *Tb*Alba2, we carried out in silico structural comparative analysis using the Phyre algorithm. The best score was obtained with the Alba protein from *Sulfolobus solfataricus*, *Sso*10b1 [35], with an E-value of 8.2e-09. The βαβαββ topology is conserved in *Li*Alba1 with an additional α helix at the C-terminal region (Fig 1B), corresponding to the previously described synapomorphic C-terminal extension containing the FDxh signature motif, which is specific of the Rpp20-like subgroup [20]. The coil regions are the most divergent in trypanosomatids but are well conserved between *Leishmania* spp. (positions 3–13, 17–22, 80–90 and 116–131 of the alignment in Fig 1B). The lysine residue usually deacetylated by Sir2 (reviewed in [36, 37]) is not conserved in trypanosomes (position 19 of the alignment) but we cannot rule out the possible acetylation on neighbor lysine residues. While the acetylation of Alba-domain proteins by Sir2 has been characterized in *Archaea* [38] and *Plasmodium falciparum* [22], there are no published reports in trypanosomatids to date. Another possible well-known posttranslational modification (PTM) is protein phosphorylation. It has been reported that *Tb*Alba2 in *T*. *brucei* bloodstream forms is phosphorylated at Serine 24 [39], an amino acid that is not conserved in *Leishmania* spp. (Fig 1B). However, in *T*. *cruzi* metacyclics, Serine 2 of Tc00.1047053504089.70 (TcCLB.504089.70) is phosphorylated and this amino acid is well conserved in *Leishmania* [40] (Fig 1B). Although the Alba-domain proteins from *L*. *infantum* have not been identified in phosphoproteome studies so far [41–43], the conservation of Ser 2 suggests that these proteins might be phosphorylated in *Leishmania*. Phosphorylation of Alba-domain proteins on Ser/Thr residues has also been reported in *Toxoplasma gondii* and *P*. *falciparum* [44–46]. In *P*. *falciparum*, phosphorylation by protein kinase CK2 [47] has been characterized for *Pf*Alba1 and 2, belonging to the Rpp20-like subgroup.

In trypanosomatids, Rpp25-like proteins have a molecular weight ranging from 20 to 25 kDa and a p*I* ranging from 9.9 to 10.3. Within *Leishmania* spp., Rpp25-like proteins share 83–96% identity, with *L*. *tarentolae* being the most divergent (83%). Sequence comparison of *Li*Alba3 with *Trypanosoma* spp. Rpp25-like proteins revealed 53–60% identity. The sequence of *Li*Alba3 shares 56% identity with the *Tb*Alba3 (Tb927.4.2040) and 53% with *Tb*Alba4 (Tb927.4.2030). Structure prediction of *Li*Alba3 using Phyre outputs Alba2 from *Aeropyrum pernix* K1 [48] as the best match (E-value 1.2e-07). The βαβαββ secondary structure seems conserved in *Li*Alba3 with an additional α helix in the N-terminal region and a longer C-terminal extension containing the RGG-box domain, which is specific of Rpp25-like proteins [20] together with the signature sequence GYQxP (position 146–150 of the alignment) (Fig 1C). As for *Li*Alba1, the coil regions are the ones with the highest divergence between *Leishmania* spp. and *Trypanosoma* spp. (positions 15–23 and 107–118 in the alignment of Fig 1C). Here, the lysine residue deacetylated by Sir2 is replaced by a threonine in *Leishmania* (position 38 of the alignment). In *P*. *falciparum*, deacetylation of *Pf*Alba3 (Rpp25-like subgroup) has been reported [21]. In *T*. *brucei* procyclic form, *Tb*Alba3 is phosphorylated at tyrosine 4 [49]. Tyrosine phosphorylation is rare in trypanosomatids and this conserved amino acid in *Trypanosoma* spp. is replaced by an arginine in all *Leishmania* species. In *T*. *cruzi* metacyclics, Ser residues 144 and 157 are phosphorylated [40], and only Ser144 is conserved in trypanosomatids, suggesting a potential phosphorylation of *Li*Alba3 on this residue.

Taken together, these sequence predictions indicate that Alba-domain proteins have a conserved structure in trypanosomatids, but *Leishmania* harbors specific mutations in key regulatory sequences shown to be phosphorylated in *T*. *brucei* or *T*. *cruzi*. The RGG-box remains also a good candidate for arginine methylation. Recent work in *T*. *brucei* and *Leishmania* showed that orthologs of *Li*Alba3 are methylated [50, 51]. In *L*. *major* and *L*. *infantum*, the RGG-box demonstrates a good conservation of the direct repeat RGGRGGS, resulting in three SR sites, a structure known to be phosphorylated in yeast and to play a role in nuclear import [52].

To look for putative PTMs in *Li*Alba3 protein, we loaded protein samples from *L*. *infantum* promastigotes and differentiating amastigotes on 2D SDS-PAGE and carried out a Western blot using the *T*. *brucei* TbAlba2040/2 antibody produced in mice [26] that specifically recognizes the *Li*Alba3 protein [18]. Interestingly, we detected 4 to 5 different forms of *Li*Alba3 in promastigotes with p*I* values ranging from 10 to 11 (S2A Fig), which suggests different levels of PTMs. During amastigote differentiation, only one prominent form was seen at p*I* 10.25 (S2B Fig).

### Alba-domain protein associated partners

**Fig 2:**
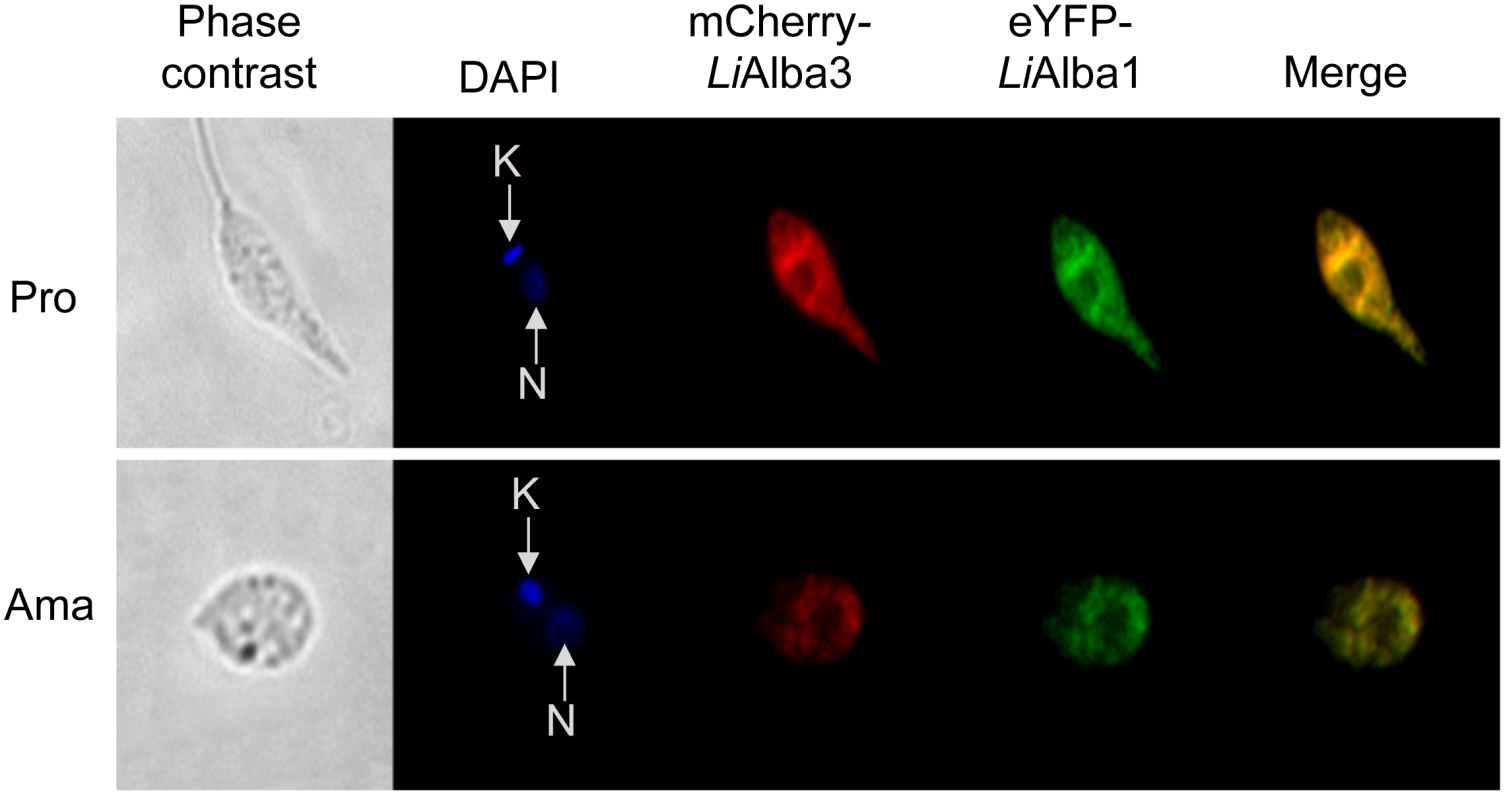
Alba-domain proteins co-localize to the cytoplasm of promastigote and amastigote *Leishmania* life stages. Direct fluorescence images of recombinant *L*. *infantum* promastigotes (Pro) and axenic amastigotes (Ama) at passage 4 co-expressing eYPF-*Li*Alba1 (green) and mCh-*Li*Alba3 (red) proteins. Green and red pixels overlapped in the digital images yielding yellow/orange signals. The nucleus (N) and kinetoplastid DNA (K) were stained with DAPI (blue).

**Fig 3:**
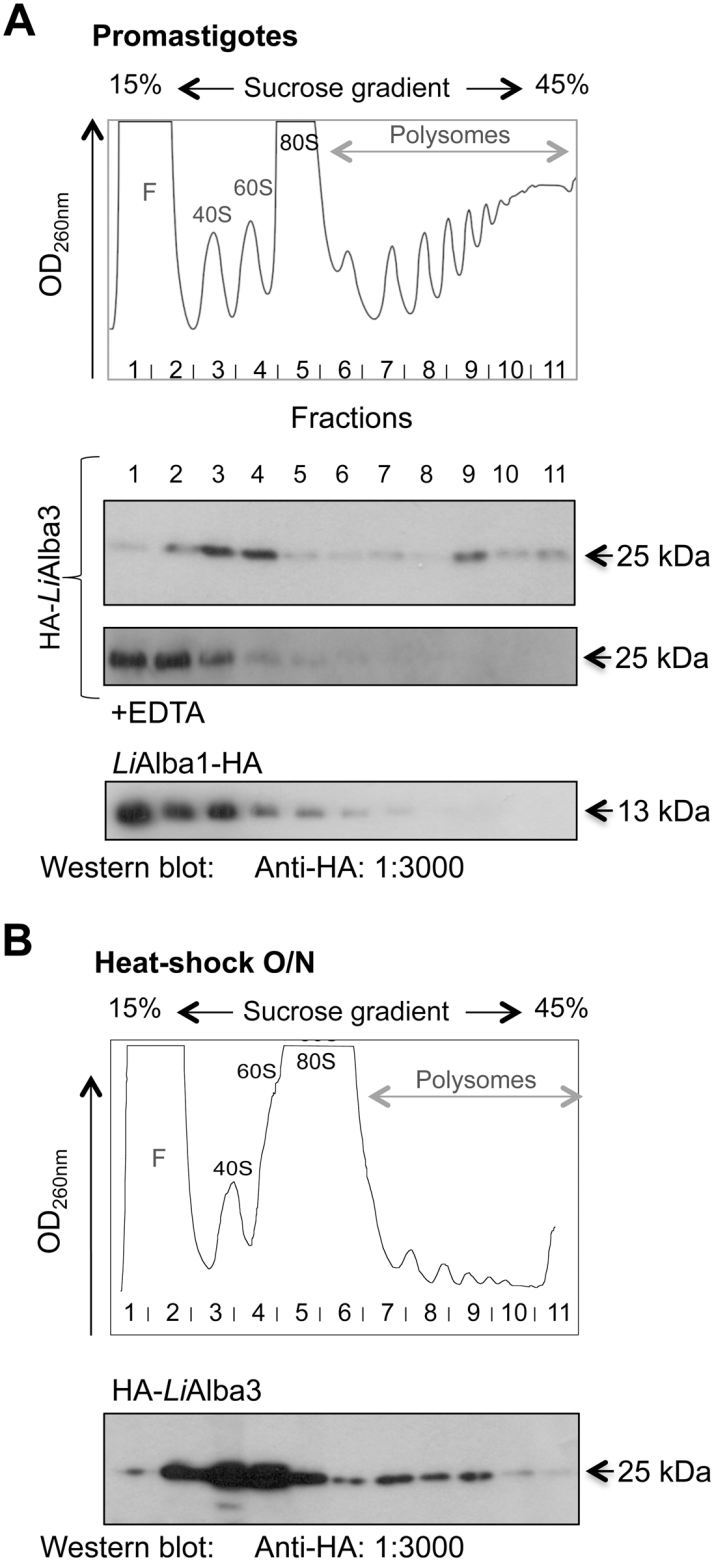
Alba-domain proteins are associated with ribosomal subunits. Polysome fractionation of *L*. *infantum* expressing HA-tagged Alba1 and Alba3 proteins by 15–45% sucrose gradient was carried out using logarithmic phase promastigotes (26°C) (A) or heat-stressed parasites grown O/N at 37°C (B). Graphical representations present the RNA content of each collected fraction after ultracentrifugation on 15–45% sucrose gradient. F: Free RNA; 40S, 60S and 80S: ribosomal subunits and monosomes, respectively. Each fraction was loaded on 12% SDS-PAGE and transferred on a nylon membrane for Western blot analysis to detect HA-*Li*Alba3 and *Li*Alba1-HA proteins using an anti-HA antibody. As a control, half of the protein extracts were incubated with EDTA before ultracentrifugation to disrupt association of the polyribosomes with mRNAs.

To get further insight into the functional role of *Li*Alba1 and *Li*Alba3 proteins, we carried out co-immunoprecipitation studies coupled with MS/MS peptide identification to characterize putative Alba-domain interacting partners. For these studies, we used *L*. *infantum* add-back strains episomally expressing the *Li*Alba1-HA or HA-*Li*Alba3 proteins into the *Li*Alba1^-/-^ and *Li*Alba3^-/-^ background, respectively (previously named *Li*Alba13^-/-^ and *Li*Alba20^-/-^, respectively in [18]) to more closely mimic endogenous levels of Alba proteins. We have shown previously [18] that episomal expression of Alba proteins in the knockout strain background resulted in similar expression levels to those of the endogenous Alba proteins. The MS/MS results from both *L*. *infantum* promastigote and axenic amastigote forms, representative of three independent experiments for each strain, are shown in Table 1. Alba1 and Alba3 are known to complex together in trypanosomes [21, 25, 53] and this interaction was also revealed in *Leishmania* both by co-immunoprecipitation (Table 1) and co-localization studies (Fig 2). This explains why most of the co-immunoprecipitated proteins were found to interact with both HA-*Li*Alba3 and *Li*Alba1-HA. *Li*Alba1 and *Li*Alba3 interact with the poly(A)-binding proteins (PABP1-3) in both parasite lifestages (Table 1 and S3A Fig) as also observed in *T*. *brucei* [25], *P*. *berghei* [23] and *T*. *gondii* [54]. Nevertheless, interaction of *Li*Alba1 and *Li*Alba3 with PABPs may be not direct as RNase A treatment prior to immunoprecipitation and Western blot analysis did not detect PABP1 (S3B Fig). *Li*Alba1 and *Li*Alba3 interact also with the DEAD-box ATP-dependent RNA helicase LinJ.32.0410, a homolog of the yeast Ded1p implicated in ribosome scanning and the distribution of mRNAs between P-bodies and polysomes [55]. We have previously shown that this RNA helicase protects rRNA from degradation under conditions of stress and cell death [32]. A 25 kDa RNA-binding protein (LinJ.32.0790) with a nucleolar RNA-binding domain specific to trypanosomatids and a ZC3H41 helicase homolog (LinJ.27.1220 [56]) were also found to interact with *Li*Alba1 and *Li*Alba3 in both developmental stages. To our knowledge, these proteins have not been studied yet. An NTF2-like protein (for Nuclear Transport Factor 2), LinJ.21.0490, interacts also with both Alba-domain proteins. In other organisms, NTF2 is involved in RanGTP transport and cargo shuttling between the cytoplasm and nucleoplasm (reviewed in [57]), and some of these factors have been studied in *T*. *brucei* for their implication in cell death [58]. Ribosomal proteins of the 40S subunit (SA, S3A, S6, S14, S24e) or 60S (L1a, L7a) were also found associated with *Li*Alba1 and *Li*Alba3 both in promastigotes and amastigotes, although a higher number of ribosomal proteins was seen in co-immunoprecipitation studies using amastigote extracts (Table 1). Association of *Li*Alba1 and *Li*Alba3 with ribosomal subunits (40S and 60S) was also suggested by sucrose gradient fractionation (Fig 3A). In the closely related species *T*. *brucei*, Alba proteins were found to co-sediment with polysomal fractions [25], but in the case of *Leishmania* they were associated predominantly with ribosomal subunits, although a small fraction was also seen in the polysomes (Fig 3A). Interestingly, under conditions of decreased global translation following O/N exposure to heat stress (Fig 3B upper panel and [59]), the enrichment of *Li*Alba3 in ribosomal subunits was significantly increased (Fig 3B, lower panel), suggesting a role of this protein in translational repression, as also reported in *Plasmodium* [23]. Higher co-fractionation of HA-*Li*Alba3 in ribosomal subunits upon heat-stress was not due to changes in protein expression, as recombinant HA-*Li*Alba3 remained stable between unstressed and heat-stressed parasites (S4C Fig). Also, no changes were observed in HA-*Li*Alba3 and *Li*Alba1-HA proteins between promastigotes and differentiating amastigotes (S4A and S4B Fig).

Proteins found to interact with *Li*Alba1 and *Li*Alba3 specifically in amastigotes include DRBD2 (LinJ.35.2240, a possible homolog of yeast Gbp2p involved in mRNA export), a homolog of Rap55 (LinJ.25.0550), a putative vacuolar protein (LinJ.19.0020 [60]), and a second NTF-2 like protein specific of trypanosomatids (LinJ.18.0300). On the other hand, a 44 kDa RNA-binding protein specific to *Leishmania* (LinJ.18.0590), a seryl-tRNA synthetase, and a dipeptidyl-peptidase III (LinJ.05.0960), mostly found in *Leishmania* spp. and metazoans, seem to interact with *Li*Alba1 or *Li*Alba3, specifically in promastigotes.

### Alba-domain proteins are localized to distinct subcellular compartments throughout *Leishmania* development

**Fig 4:**
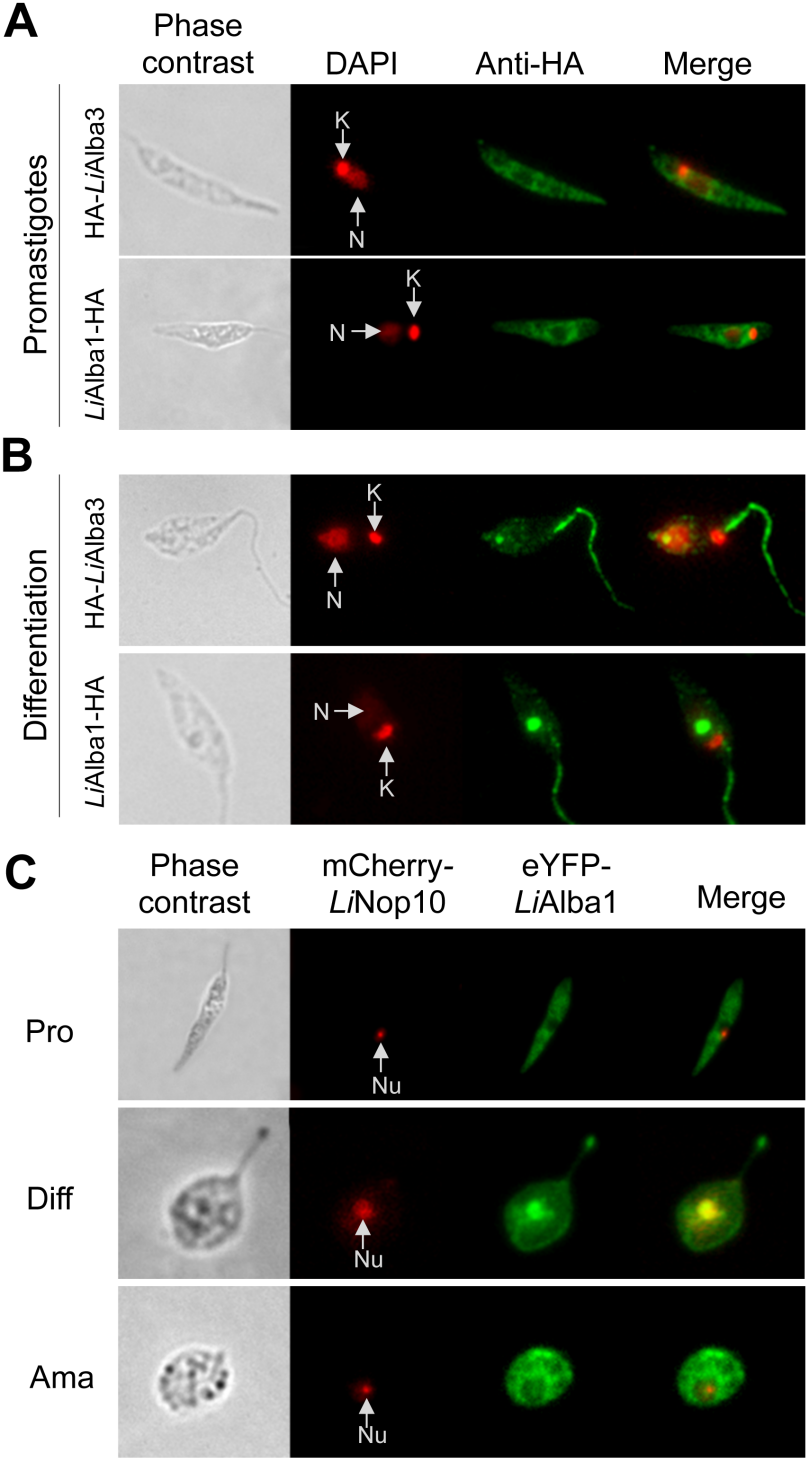
Alba-domain proteins translocate from the cytoplasm to the flagellum and the nucleolus upon *Leishmania* amastigote differentiation. Subcellular localization of *Li*Alba1-HA and HA-*Li*Alba3 proteins in promastigotes (A) and upon amastigote differentiation (8 h in MAA medium pH 5.8 at 37°C) (B) was assessed by indirect immunofluorescence studies using an anti-HA antibody as described in Materials and Methods. DAPI staining (red) allows detection of the nucleus (N) and kinetoplastid DNA (K). C) Immunofluorescence images of wild type *L*. *infantum* episomally co-expressing pSP-NEOalphaIR-eYPF-*Li*Alba1 and pSP-HYGalphaIR-mCh-*Li*NOP10 grown as promastigotes (Pro), differentiating amastigotes (Diff) and amastigotes (Ama). *Li*NOP10 was used as a nucleolar (Nu) control.

Fluorescent protein imaging studies using *L*. *infantum* promastigotes and axenic amastigotes expressing either eYFP-*Li*Alba1 or mCh-*Li*Alba3 demonstrated that *Li*Alba1 and *Li*Alba3 proteins co-localize to the cytoplasm of both major parasite lifestages (Fig 2), as also described in *T*. *brucei* [25, 26] and consistent with a role of these proteins in RNA metabolism. In other organisms as plants, Alba-domain proteins are described as stress responsive proteins [61, 62] and in *T*. *brucei* they accumulate in starvation granules [25]. Therefore, we investigated the subcellular localization of Alba-domain proteins upon amastigote differentiation triggered in vitro by exposure of promastigotes to combined heat stress (25°C to 37°C) and acidic environment (from neutral to acidic pH). We used the add-back strains previously described [18] where HA-tagged versions of *Li*Alba1 and *Li*Alba3 were episomally expressed into the *Li*Alba1^-/-^ and *Li*Alba3^-/-^ background, respectively. Expression of recombinant HA-*Li*Alba3 and *Li*Alba1-HA proteins remained stable between promastigotes and differentiating amastigotes (S4A and S4B Fig). Immunolocalization studies confirmed the cytoplasmic co-localization of *Li*Alba1-HA and HA-*Li*Alba3 proteins in promastigotes (Fig 4A), in agreement with data obtained in Fig 2 using fluorescent Alba proteins. The cytoplasmic localization of Alba proteins in promastigotes is not homogenous (Figs 2 and 4A), suggesting that they may associate with subcellular compartments. Digitonin fractionation of *L*. *infantum* promastigotes showed that native *Li*Alba3 (S5A Fig) as well as HA-tagged *Li*Alba3 (S5B Fig) and *Li*Alba1 proteins (S5C Fig) were not only enriched in fraction 2 (mainly cytosolic proteins) but also in fraction 3 (enriched for organelle-located proteins) [63]. To rule out the possibility that Alba proteins associate with the endoplasmic reticulum or the mitochondrion, we carried out a co-immunolocalization studies with BiP, an ER chaperone, and MitoTracker staining, respectively. Our results indicate that *Li*Alba proteins do not associate with the endoplasmic reticulum (S6A Fig) or the mitochondrion (S6B Fig).

Interestingly, upon amastigote differentiation (from 8 h up to overnight exposure to the differentiation signals), the vast majority of Alba1 and Alba3 proteins were not cytoplasmic but were enriched inside the nucleus and all along the flagellum (Fig 4B and data not shown). During differentiation, Alba proteins were also enriched in fraction 5 corresponding to insoluble or membrane-associated material as shown by digitonin fractionation and Western blot experiments (S5 Fig, right panels). Within the nucleus, *Li*Alba proteins accumulate in a region with a weaker DAPI staining (data not shown), usually corresponding to the nucleolus as described previously [64, 65]. We therefore investigated whether Alba proteins were localized to the nucleolus. As a control, we used the Nop10 protein (S7 Fig), previously characterized as a nucleolar component in *T*. *brucei* [66]. To confirm nucleolar localization of Alba proteins upon differentiation, we generated a recombinant strain episomally co-expressing eYFP-*Li*Alba1 and mCh-*Li*Nop10 proteins in the wild type background. A co-localization signal with *Li*Nop10 confirmed the presence of *Li*Alba proteins in the nucleolus (Fig 4C). It is noticeable that during differentiation the *Li*Nop10 signal is stronger and this could correlate with nucleolar rearrangements during stress in eukaryotes (reviewed in [67, 68]). Even if some parasites show only nucleolar or only flagellar localization of Alba proteins, it is experimentally difficult to obtain a clear chronological order of these events. The flagellar and nucleolar localization of Alba proteins is detected only upon amastigote differentiation and the proteins are relocalized back to the cytoplasm in fully differentiated amastigotes (Fig 2). Differential localization of Alba proteins is a regulated process as when differentiating amastigotes were switched back to promastigote growth conditions, the nucleolar and flagellar signals were lost, and Alba proteins were seen again predominantly in the cytoplasm (S8 Fig).

To determine if *Li*Alba proteins form different complexes upon amastigote differentiation, hence explaining their differential localization, we carried out co-immunoprecipitation experiments with parasites grown O/N under conditions of amastigote differentiation *in vitro*. These experiments did not reveal any new Alba protein associations, which could explain translocation of Alba proteins to the nucleolus and the flagellum (data not shown).

### Heat-shock triggers translocation of Alba-domain proteins from the cytoplasm to the nucleolus and the flagellum

**Fig 5:**
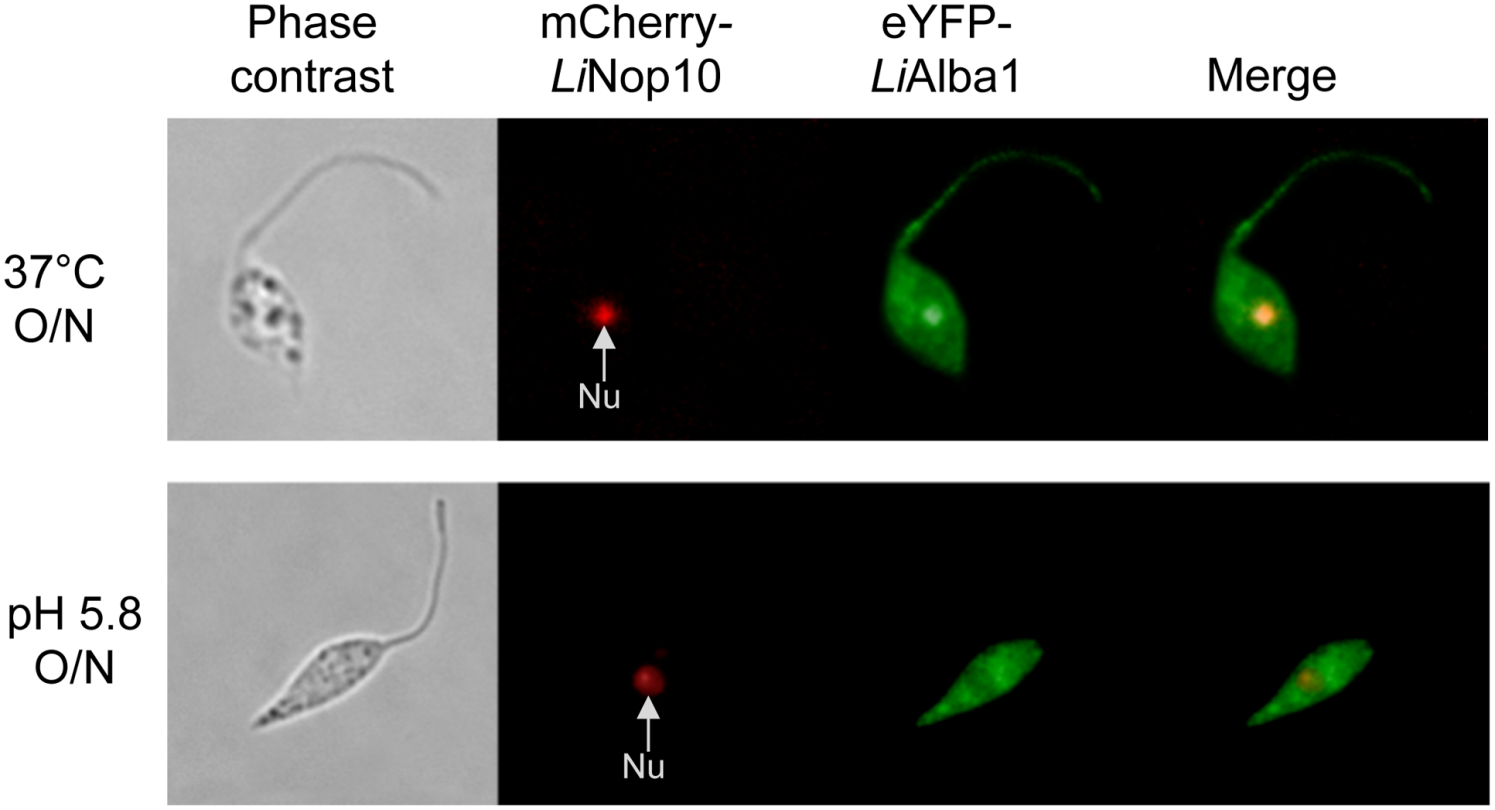
Heat stress triggers differential localization of Alba-domain proteins in *Leishmania*. Immunofluorescence images for eYPF-*Li*Alba1 (green) and mCh-*Li*NOP10 (red) in *L*. *infantum* promastigotes co-expressing pSP-NEOalphaIR-eYPF-*Li*Alba1 and pSP-HYGalphaIR-mCh-*Li*NOP10 submitted to heat stress (from 25°C to 37°C) or to acidic pH (pH 5.8) O/N. Nu: nucleolus.

To investigate further the differentiation signal(s) necessary for Alba protein translocation into the nucleolus and the flagellum, we exposed *L*. *infantum* promastigotes to either temperature stress (from 25° to 37°C in SDM medium pH 7.0 O/N) or acidic pH (pH 5.8 in HCl acidified SDM medium at 25°C O/N). Temperature stress and acidic pH represent the most important triggering factors of *Leishmania* differentiation into amastigote forms inside macrophages [4]. Temperature stress was sufficient for *Li*Alba1 protein translocation into the nucleolus and the flagellum (Fig 5, upper panel). Similar results were obtained with *Li*Alba3 (data not shown). In contrast, pH stress did not have any effect on Alba protein differential localization (Fig 5, lower panel).

In a time course experiment, we evaluated the kinetics of Alba protein flagellar localization upon exposure to temperature stress using microscopic examination. The *Li*Alba3^-/-^ knockout strain episomally expressing mCh-Alba3 was used for these experiments. Parasites with Alba3 localized to the flagellum were observed 2 hours following temperature stress and after 8 hours more than 26% of the total population showed this pattern (S9 Fig). Exposure of parasites to temperature stress can induce, under certain conditions, parasite killing [69]. Therefore, we investigated whether Alba protein localization in the nucleolus or the flagellum is associated with dying cells. Using flow cytometry, we compared the percentage of cells having a flagellar signal with the percentage of cells in SubG1 population, representative of dead cells with nuclei containing degraded DNA. During the 8 h period of temperature stress, the proportion of SubG1 population remained stable ranging from 2% to 2.5% (S9 Fig), a value clearly inferior to the 26% of parasites showing Alba3 flagellar localization (S9 Fig). Thus, these data exclude any correlation between flagellar localization of *Li*Alba3 and cell death.

Inhibition of transcription using Actinomycin D is another type of stress previously reported in *Leishmania* and also in *T*. *cruzi* to induce nucleolar accumulation of cytoplasmic RNA-binding proteins [65]. We therefore tested whether transcriptional stress induces Alba protein accumulation in the nucleolus but we did not observe any of this upon Actinomycin D treatment (data not shown).

### Partial characterization of the flagellar proteome of *Leishmania* promastigotes and differentiating amastigotes

**Fig 6:**
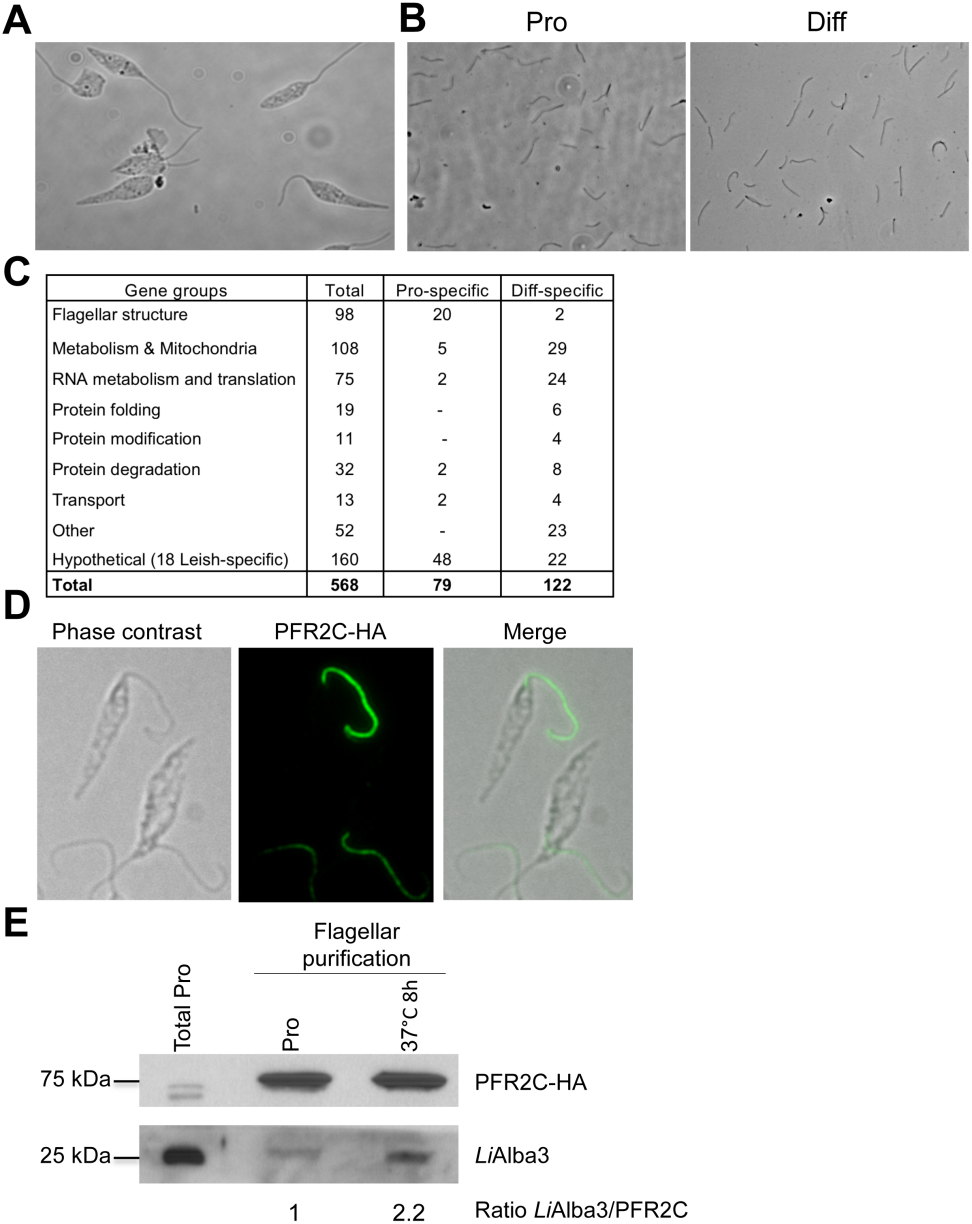
Flagellum purification demonstrates an enrichment of *Li*Alba3 in the *Leishmania* flagellum during heat stress. Phase contrast images of intact *L*. *infantum* promastigotes (A) and of purified flagella after sucrose gradient isolation (B) as described in Materials and Methods. (C) Summary of MS/MS identified proteins from four independent experiments of flagellum purification (two from promastigote cell extracts and two from 8 h-differentiating amastigotes). Identified genes were classified according to their gene ontology and to characterized orthologs in *Trypanosoma* spp. based on GeneDB and TriTrypDB gene annotations. Only genes identified at least twice with a minimum of 2 peptides are shown here (see Tables 2, S3 and S4 for the complete list of the identified *L*. *infantum* flagellum proteins). (D) Confirmation of flagellar localization of PFR2C-HA in recombinant *L*. *infantum* expressing pSP-alphaIRNEOalphaIR-PFR2C-HA. (E) Western blot analysis and quantification of endogenous *Li*Alba3 in purified flagellum fractions upon promastigote conditions of growth (Pro) or following 8 h of temperature stress (37°C). After flagellum purification, flagella from promastigotes and heat-stressed parasites were counted on a Malassey (hemocytometer) to load an equivalent number of flagella on the gel. Total proteins of promastigote cells were loaded as a control. Relative quantification was performed using ImageJ blot.

The presence of RNA-binding proteins such as Alba proteins in the *Leishmania* flagellum is an intriguing finding. We therefore carried out additional experiments to confirm by other means the presence of Alba proteins in the flagellum and to examine if other RNA-binding proteins were also present in this organelle. For this purpose, we adapted a recently published protocol in *T*. *brucei* [30] to purify intact flagella from wild type *L*. *infantum* promastigotes and parasites undergoing amastigote differentiation (8 h exposure to differentiation signals) (Fig 6A and 6B) and analyzed this material by mass spectrometry. S3 Table lists all the components of the *Leishmania* flagellum proteome of promastigotes and also of differentiating amastigotes identified in this study and S4 Table provides the detailed peptide and spectrum reports of these experiments. Ninety-eight genes coding for various proteins previously described to be associated with the flagellum were identified here, including components of the paraflagellar rod (15 proteins) and the axoneme such as radial spoke proteins (8 proteins), components of the dynein arm (7 proteins) and other proteins (17 proteins) conserved in species with motile flagella (CMF, components of motile flagella) [70]. In addition, proteins normally found in the membrane or matrix fraction of flagella were detected such as intraflagellar transport proteins [71], SMP-1 [72], FLAM7 and 14-3-3 protein [30] or flabarin [73] (Fig 6C, Tables 2 and S3). Seventy-five proteins involved in RNA metabolism and translation, 108 proteins participating in mitochondrial function and metabolic processes, proteins involved in protein folding (19 proteins), post-translational modifications (11 proteins), protein degradation (32 proteins), and intracellular transport (13 proteins) were also identified as part of the *Leishmania* flagellum proteome (Fig 6C, Tables 2 and S3). Table 2 summarizes the best-characterized proteins belonging to flagellar structures and RNA metabolism found in our experiments. *Leishmania* shares 57% of the identified proteins in common with the flagellum proteome of *T*. *brucei* procyclics characterized using a similar strategy [30]. The most important differences between these two related parasitic protozoa include components of the proteasome machinery seen only our *Leishmania* flagellar fraction (17 in total) and 18 proteins that are specific to *Leishmania*.

**Table 2:** List of the most representative components of the flagellar structure and RNA metabolism enriched in the *L*. *infantum* flagellum proteome.

	Gene ID	Identified Proteins	MWkDa	Pro (No. peptides)	Diff 8h (No. peptides)
**Paraflagellar rod components**	LinJ.29.1880 (+1)	paraflagellar rod protein 1D, putative	69	41–19	16–19
	LinJ.16.1510	paraflagellar rod protein 2C	69	32–21	17–23
	LinJ.36.4440	paraflagellar rod component, putative	123	20–10	1–6
	LinJ.09.1390	paraflagellar rod component, putative	68	14–5	7–8
	LinJ.07.0470	paraflagellar rod component, putative (PFC3) | *Tb*	89	21–4	3–6
	LinJ.36.6130	paraflagellar rod component, putative	86	15–10	2–9
	LinJ.05.0040	paraflagellar rod component par4, putative	68	12–3	2–3
	LinJ.02.0280	paraflagellar rod component, putative (PFC11) | *Tb*.	73	7–2	0–3
	LinJ.27.1750	paraflagellar rod protein-like protein	89	10–1	0–3
	LinJ.19.0520	paraflagellar rod component, putative (PFC15) | *Tb*	58	7–2	0–4
	LinJ.36.5010	paraflagellar rod component, putative	34	4–3	0–3
**Components of motile flagella**	LinJ.27.0720	hypothetical protein, conserved | *Tb*CMF2	85	22–11	9–14
	LinJ.08.1030	hypothetical protein, conserved | *Tb*CMF3	97	21–4	2–2
	LinJ.28.1210	hypothetical protein, conserved | *Tb*CMF15	68	11–6	3–2
	LinJ.13.1580	hypothetical protein, conserved | *Tb*CMF22	104	12–5	0–4
	LinJ.30.3700	hypothetical protein, conserved | *Tb*CMF46	64	11–2	1–3
	LinJ.27.2090	hypothetical protein, conserved | *Tb*CMF5	174	9–7	0–0
**Radial spoke components**	LinJ.13.0290	flagellar radial spoke protein, putative	67	10–10	4–5
	LinJ.08.0900	radial spoke protein RSP2, putative | *Tb*	63	13–2	3–4
	LinJ.27.2530	radial spoke protein 3, putative	42	6–4	5–3
	LinJ.33.2630	radial spoke protein RSP9, putative	36	7–2	3–3
	LinJ.03.0950	radial spoke protein RSP10, putative | *Tb*.	36	9–4	1–3
	LinJ.29.0690	flagellar radial spoke protein-like, putative	76	5–3	0–1
**Actin & Tubulin**	LinJ.08.1290 (+1)	beta-tubulin	50	28–25	27–24
	LinJ.13.0330 (+1)	alpha-tubulin	50	34–20	21–20
	LinJ.04.1250	actin	42	7–3	1–3
**Dyneins**	LinJ.25.1010	dynein heavy chain, putative	537	22–6	1–18
	LinJ.13.1390	dynein heavy chain, putative	529	17–8	0–12
	LinJ.32.1120	dynein, putative	68	4–4	4–6
	LinJ.24.0270	dynein intermediate-chain-like protein	75	8–3	1–3
	LinJ.32.3050	outer dynein arm docking complex protein	70	5–3	0–6
	LinJ.33.2770	dynein intermediate chain, putative	115	10–3	0–4
	LinJ.26.1100	dynein arm light chain, axonemal, putative | *Tb*	50	8–3	1–1
**Other flagellar components**	LinJ.05.1080	hypothetical protein, conserved | axoneme *Tb*	80	13–8	6–9
	LinJ.14.1300	hypothetical protein, conserved | Flagellum *Tb*	99	22–7	0–4
	LinJ.11.0810	hypothetical protein, conserved | axoneme) *Tb*	104	10–7	5–9
	LinJ.20.1350	small myristoylated protein-1, putative	15	1–4	4–3
	LinJ.24.2060	STOP axonemal protein, putative	30	10–4	4–1
	LinJ.20.1450	axoneme central apparatus protein, putative	55	5–5	2–7
	LinJ.10.1280	flagellar protofilament ribbon protein-like protein	47	14–3	1–0
	LinJ.32.4020	myosin XXI	119	8–1	2–6
	LinJ.32.3350	hypothetical protein, conserved | FLAM7	292	10–7	0–6
	LinJ.31.1240	vacuolar-type proton translocating pyrophosphatase 1	83	7–4	5–4
	LinJ.35.5310	hypothetical protein, conserved	39	6–3	2–4
	LinJ.17.0970	META domain containing protein, putative	48	8–1	1–3
	LinJ.29.2310	GTP-binding protein, putative | dynamin	78	3–0	9–0
**RNA metabolism**	LinJ.01.0790 (+1)	eukaryotic initiation factor 4a, putative	45	7–8	10–9
	LinJ.32.0410	ATP-dependent RNA helicase, putative	67	16–8	17–12
	LinJ.21.1820	RNA helicase, putative	59	4–2	6–4
	LinJ.35.0370	ATP-dependent DEAD-box RNA helicase, putative	46	3–0	6–6
	LinJ.35.3150	ATP-dependent RNA helicase, putative	101	2–0	7–4
	LinJ.07.0130	ATP-dependent DEAD/H RNA helicase, putative	53	0–0	4–3
	LinJ.35.4200	poly(A)-binding protein 2, putative	65	5–0	7–2
	LinJ.25.0080	poly(A)-binding protein 3, putative	61	1–0	2–0
	LinJ.35.2240	RNA binding protein, putative	30	1–0	2–2
	LinJ.34.2870	ZFP family member, putative (ZC3H10) | *Tb*	64	4–3	1–2

Proteome analyses were performed in duplicate for *L*. *infantum* promastigotes and differentiating amastigotes (8 h). Only proteins identified with an average of 2 peptides in 2 independent experiments and a probability of >95.0% to correspond to the correct protein were included here. A more complete list of identified proteins (with 2 peptides in at least one experiment) is presented in S3 Table. The raw data of the peptide and spectrum reports are provided in S4 Table. Because *Leishmania* databases are still poorly annotated for flagellum components, we also analyzed the *Trypanosoma* orthologs to include proteins previously described as flagellar components [30] (see gene names or comments on TritrypDB.org). The reference to the *T*. *brucei* (*Tb*) orthologs is shown by the presence of the | separator.

Several RNA-binding proteins were found in the *Leishmania* flagellar fraction, including several ATP-dependent RNA helicases, translation initiation factors, and zinc-finger domain proteins (Tables 2 and S3). Only a single peptide for *Li*Alba1 was identified under differentiation in two immunoprecipitation experiments (data not shown). This result could be explained if the amount of Alba proteins in the flagellum was too low relative to the major skeletal components such as those in the PFR or the axoneme. However, two amastin surface proteins were found in the flagellum of differentiating amastigotes (S3 Table). We have shown recently that Alba-domain proteins regulate the stability of *amastin* transcripts [18].

To further confirm the presence of Alba-domain proteins in the flagellar fraction, the experiment was repeated with extracts from promastigotes and parasites exposed for 8 hours to heat stress. As a control for flagellar localization, we used recombinant parasites episomally expressing the paraflagellar rod gene *PFR2C* tagged with an HA epitope at the C-terminus (PFR2C-HA). Specific localization of this protein in the flagellum was shown by immunofluorescence (Fig 6D). After flagellum purification, the samples were used for Western blot analysis against PFR2C-HA (control) and endogenous Alba3 antibodies in parallel (Fig 6E). This purification strategy indicated that *Li*Alba3 is enriched by 2.2-fold in the flagellar fraction of *L*. *infantum* parasites exposed to heat stress (Fig 6E), hence confirming the immunofluorescence data in Figs 4B and 5.

Interesting differences were observed in the composition of the flagellar fraction proteome between *L*. *infantum* promastigotes and differentiating amastigotes. For example, the flagellum of parasites undergoing differentiation lacks 2 paraflagellar rod components, 5 dyneins, 1 radial spoke protein (RSP11), 2 flagellar transport proteins, and few (10) other proteins associated with flagellum functions. There is also a large number of hypothetical proteins and most of the hypothetical *Leishmania*-specific proteins (48 in total) that were not detected in the flagellum of differentiating amastigotes (Fig 6C and S3 Table). On the other hand, several proteins involved in protein degradation, protein folding, intracellular protein transport, GTP/ATP binding, cell-redox homeostasis, and oxidation-reduction processes were mostly detected in the flagellum of differentiating parasites (S3 Table). In total, 79 proteins were enriched in the promastigote flagellum and 122 in that of differentiating amastigotes (Fig 6C). Although the length of the flagellum between promastigotes and differentiating parasites was relatively similar (data not shown) and co-immunoprecipitation studies were carried out under the same conditions, additional quantitative proteomic studies are needed to conclude about differences in flagellum composition between promastigotes and differentiating amastigotes.

## Discussion

Recent studies in protozoan parasites like *Plasmodium* [23], *Toxoplasma* [54], *Trypanosoma* [25], and *Leishmania* [18] highlight the role of Alba-domain proteins in gene regulation at the interface between mRNA stability and translation initiation. In our previous work, we showed that *Li*Alba3 contributes to the stabilization of *delta-amastin* transcripts, specifically in the amastigote stage, suggesting different roles of the protein during the parasite life cycle [18]. Here, we focused on the molecular and cellular characterization of Alba-domain proteins in *Leishmania*. We identified similarities but also important differences between Alba protein members in parasitic protozoa. In contrast to *Trypanosoma* species that possess four Alba-domain proteins, *Leishmania* species have only two Alba proteins, representative of the Rpp20- and the Rpp25-like eukaryotic subgroups respectively. Pull-down experiments of epitope tagged *Li*Alba proteins coupled to MS/MS peptide identification revealed interactions between *Li*Alba1 and *Li*Alba3 but also their association with components of the translation machinery, such as ribosomal proteins of the 40S and 60S subunits and all three poly(A)-binding proteins. These interactions and the enrichment of Alba proteins in the ribosomal subunit fractions suggest a role of these proteins in translation initiation, as also reported for *T*. *brucei* [25]. Alba proteins were previously described as components of a translationally silent complex containing stage-regulated mRNAs in *Plasmodium* [23]. Here, we show that Alba-domain proteins are highly enriched in ribosomal subunits under stress where global translation is drastically reduced, also suggesting a possible role of these proteins in translational repression.

Our immunofluorescence studies showed that both *Li*Alba proteins co-localize mostly to the cytoplasm of promastigote and amastigote lifestages. However, upon amastigote differentiation, Alba proteins translocate from the cytoplasm to the nucleolus and the flagellum. Flagellar localization of Alba proteins was also suggested using flagellum purification strategies in addition to immunofluorescence studies. Heat-shock from 25°C to 37°C, known to trigger amastigote differentiation [4], is sufficient and necessary for Alba protein translocation. The flagellar and nucleolar localization of Alba proteins is transient, as they are found back to the cytoplasm once the differentiation process is completed, suggesting that shuttling of Alba proteins is a regulated process. At this stage of investigation, it is not clear whether localization of Alba proteins to the nucleolus and the flagellum is the result of independent or coordinated events. Differential localization of Alba proteins has been reported previously in other protozoan parasites. For example, in intracellular forms of *T*. *gondii*, *Tg*Alba proteins are mainly cytoplasmic but accumulate in the nucleus and in perinuclear spots in extracellular parasites [54]. In *T*. *brucei* bloodstream forms, the *Tb*Alba proteins were identified as part of the flagellum-specific matrix proteome [74]. In *T*. *brucei* procyclics, the four *Tb*Alba are cytoplasmic [25] but upon starvation stress *Tb*Alba3 and *Tb*Alba4 co-localize with the RNA-interacting protein DHH1 in granules [26]. We did not detect any interactions of Alba proteins with DHH1 by immunoprecipitation studies but we found that the Rap55 homolog, a well-known partner of DHH1, interacts with Alba proteins in *Leishmania*.

Translocation of Alba proteins from the cytoplasm to both the nucleolus and the flagellum during amastigote differentiation is of particular interest. In eukaryotes, the primary function of the nucleolus is the biogenesis of ribosome subunits, including rRNA maturation (reviewed in [75]). However, the nucleolus has also been implicated in stress response (reviewed in [67]). In *L*. *mexicana* and *T*. *cruzi*, but not in *T*. *brucei*, heat stress or transcriptional stress upon Actinomycin D treatment promotes nucleolar accumulation of poly(A)^+^ mRNAs and various RNA-binding proteins such as *Tc*SR62 (mRNA stability and processing), *Tc*PABP1 and *Lmex*PABP2, *Tc*PTB2 (polypyrimidine tract binding protein), *Tc*SF3b155 (splicing factor), and *Tc*FIP1 (involved in polyadenylation) [65, 76]. However, in the case of *L*. *infantum*, Actinomycin D treatment did not induce any accumulation of *Li*Alba proteins in the nucleolus (data not shown). Because Alba-domain proteins are constituents of the RNase P/MRP in higher eukaryotes, *Li*Alba may play a role in tRNA or rRNA processing during differentiation. Global translation is downregulated upon stress and differentiation in *Leishmania* [59], and Alba proteins may contribute to this process as supported by the higher co-sedimentation of these proteins with ribosomal subunits upon conditions of decreased translation. It has been shown recently that components of the *T*. *brucei* rRNA processing machinery have an additional role in regulating mRNAs transcribed in the nucleolus by RNA polymerase I, such as GPEET [77]. In response to stress, RNA-binding cytoplasmic proteins can relocate to the nucleus. For example, transcription inhibition with Actinomycin D causes accumulation of *Lm*PABP2 and *Lm*PABP3 in the nucleoplasm [12], and arsenite stress results in the nuclear accumulation of *Tc*UBP-1 protein [78].

The flagellum is a multifunctional organelle that not only contributes to the parasite motility but also plays important roles in cell morphogenesis and host-parasite interactions [79, 80]. As the flagellum does not possess its own translation machinery, all its constituents are synthesized and transported from the cytosol [81]. During amastigote differentiation, several changes operate in the expression of stage-specific flagellum components and particularly in the length of flagellum, which becomes a rudimentary organelle, very short and non motile in amastigote forms (reviewed by [79]). The flagellar localization of Alba proteins during the first hours of amastigote differentiation might be a way to sequester these proteins together with some of their mRNA targets, hence protecting them from degradation or alternatively taking them out of the translation machinery. Sequestration of Alba proteins to the flagellum could also prevent their binding to other proteins or RNP complexes regulating (positively or negatively) the expression of specific gene subgroups required only during amastigote differentiation. We have shown previously that amastigote-specific transcripts such as *delta-amastins* and *A2* are stabilized by *Li*Alba3 [18]. RNA immunoprecipitation and RNA sequencing could be interesting follow-up studies to identify RNA species interacting with Alba proteins during the differentiation process. It is reasonable to hypothesize that Alba proteins are translocated to the flagellum through their association with other proteins. However, co-immunoprecipitation experiments upon amastigote differentiation did not reveal any new interacting partners of *Li*Alba3 that could explain changes in its subcellular localization (data not shown). Post-translational modifications could alter interactions of the Alba proteins with their mRNA targets or partner proteins and thus interfere with their differential localization. Consistent with this possibility is our finding that *Li*Alba3 is post-translationally modified in promastigotes, possibly through methylation in one of the RGG motifs, but not during amastigote differentiation. PTMs on Alba proteins could also take place within the flagellum as described for other regulatory proteins in primary cilium (reviewed in [82]).

Here, we have also partially characterized, for the first time, the flagellum proteome of *Leishmania* promastigotes and differentiating amastigotes using intact flagella preparations. Five hundred sixty eight proteins were identified as part of the *Leishmania* flagellum-enriched fraction. Approximately 57% of these proteins are in common with the recently characterized *T*. *brucei* flagellum proteome [30] but only 30% of similarities were detected when compared with the flagellar proteome of bloodstream procyclics established using a different approach [74]. In addition to ~100 flagellum constituents, including proteins of paraflagellar rod, radial spoke, dynein complex and axoneme, the *Leishmania* flagellum fraction contains a number of chaperones/chaperonins, ribosomal proteins and translation factors, components of the mitochondrial oxidative phosphorylation machinery, proteins involved in metabolism and transport, components of the proteasome, and a large number of hypothetical proteins, some of which are *Leishmania*-specific. The presence of mitochondrial proteins in the flagellum is not surprising given the strong association between the kinetoplast and the flagellum around the flagellum attachment zone. However, the fact that our flagellar preparations were DAPI positive could suggest a contamination with mitochondrial fractions. In addition to Alba-domain proteins, other RNA-binding proteins were also found in the *Leishmania* flagellum-enriched fraction. Interestingly, some of these RNA-binding proteins (PABP2, PABP3, ATP-dependent RNA helicase LinJ.32.0410, LinJ.32.0790, LinJ.27.1220, and LinJ.35.2240) have been identified in *Li*Alba pull-downs. The putative dipeptidyl-peptidase III (LinJ.05.0960), which plays a role in protein turnover and is responsive to different stresses in other organisms [83], and the trypanosomatid ortholog of NTF-2 (LinJ.21.0490) are also enriched in the flagellum fraction, and interestingly these proteins seem to interact with *Li*Alba3 by co-immunoprecipitation assays. NTF-2 might act as a cargo allowing translocation of *Li*Alba proteins to the flagellum and it was only seen in the flagellum of differentiating amastigotes. Unfortunately, it was not possible to obtain a viable null mutant of the *NTF-2* gene for further studies (data not shown). Some flagellar components (20 proteins) and several hypothetical proteins, including most of the *Leishmania*-specific ones (48 in total) were missing from the flagellum preparation of differentiating amastigotes. On the contrary, several proteins involved in metabolism and mitochondrial function (29 in total), RNA metabolism and translation (24 proteins), protein folding and degradation were enriched in the flagellar fraction of differentiating parasites. In total, 201 proteins were found preferentially in the flagellum of either promastigotes or differentiating amastigotes. Nevertheless, additional experiments are required to confirm these differences.

Shuttling of Alba-domain proteins between the cytoplasm and the nucleolus or the flagellum upon *Leishmania* promastigote to amastigote differentiation underlines an important role(s), yet to be elucidated, that these RNA-binding proteins may play in regulating gene expression during the parasite development.

## Supporting Information

S1 Fig Schematic representation of the expression vectors made for this study. (A). The strategy we used for episomally expressing eYFP-*Li*Alba1, mCh-*Li*Alba3, and mCh-*Li*Nop10 fluorescent proteins or for their genomic integration is the same for the three vectors. The fluorescent coding sequence (e.g. mCherry, and yellow fluorescent protein (eYFP)) was fused at the N-terminal part of *Li*Alba1, *Li*Alba3 or *Li*Nop10 to keep the natural context of 3’UTR, hence allowing efficient regulation of the transcripts. The first cassette harboring a selectable marker gene (*NEO* or *HYG*), the alpha-tubulin intergenic region (alphaIR) for optimal processing, and the fluorescent protein without the stop codon was prepared by Phusion PCR. The NEO-alphaIR-eYFP and HYG-alphaIR-mCh cassettes were cloned into pGEM-T Easy vector for sequencing and further sub-cloning. The second step consisted in the amplification of a 500 bp fragment corresponding to the 5’-intergenic region of the target gene and the ORF together with the corresponding 3’-intergenic region (~500 bp). These two fragments were fused by PCR and an EcoRV site was added at the junction. (B) The mCh-*Li*Alba3 cassette was subcloned into pSP-alphaIRNEOalphaIR XbaI and NdeI sites to generate vector pSP-alphaIRNEOalphaIR-mCh-*Li*Alba3. PFR2C-HA was produced by PCR amplification and subcloned into pSP-alphaIRNEOalphaIR vector to generate pSP-alphaIRNEOalphaIR-PFR2C-HA. The primers used for PCR amplifications are listed in S1 Table.
(TIF)

S2 Fig 2D-gel analysis to identify post-translationally modified forms of *Li*Alba3. Total proteins from *L*. *infantum* promastigotes (A) or stationary promastigotes subjected to amastigote differentiation for 4 h (B) were loaded on a 2D gel (pH 6–11) and transferred on a nylon membrane for Western blotting using the *T*. *brucei* specific antibody recognizing the endogenous *Li*Alba3. The “smiling effect” is probably due to the migration and not to an increase in the molecular weight of *Li*Alba3 protein, as only a 25 kDa band was observed on 1D gels. p*I* between 10 and 11 is indicated on the top.
(TIF)

S3 Fig Alba protein co-immunoprecipitation controls. (A) Western blots of the HA-*Li*Alba3 pull-downs to confirm Alba-poly(A) binding protein (PABP) interactions under conditions of promastigote (Pro) or amastigote (Ama) growth or 6 h following amastigote differentiation (Diff) using antibodies made against the *T*. *brucei* PABP1-3 proteins that recognize the *Leishmania* othologs (antibodies kindly provided by Dr. Osvaldo de Melo Neto, Recife, Brazil; da Costa Lima TD *et al*., Eukaryot Cell 2010, 9(10):1484–94). (B) Western blot analysis of RNase A-treated samples prior to *Li*Alba1-HA co-immunoprecipitation using an antibody against PABP1 (upper panel) and an anti-HA antibody to detect *Li*Alba1-HA protein (lower panel).
(TIF)

S4 Fig Alba protein expression remains stable during *Leishmania* amastigote differentiation and under heat stress. Western blot analysis using an anti-HA antibody to assess expression levels of the recombinant *Li*Alba1-HA (A) and HA-*Li*Alba3 (B) proteins during promastigote (pro) to amastigote (ama) differentiation (diff) or under heat stress (O/N) (C). An antibody against alpha-tubulin was used as loading control.
(TIF)

S5 Fig Digitonin fractionation analysis of *L*. *infantum* expressing *Li*Alba1-HA and HA-*Li*Alba3 proteins. *L*. *infantum* wild type cells (WT) (A) and *L*. *infantum* add-back strains expressing HA-*Li*Alba3 (B) and *Li*Alba1-HA (C) into the *Li*Alba3^-/-^ and *Li*Alba1^-/-^ background, respectively, grown as promastigotes (exponential phase) or upon conditions of amastigote differentiation (MAA medium at pH 5.8 and 37°C for 8h) were used for these studies. Digitonin fractionation was done as previously described [63] and fractions from 1 to 5 were loaded on SDS-PAGE and transferred for Western blot analysis using specific antibodies against *Tb*Alba3, HA, and alpha-tubulin. 1 and 2 correspond to cytosolic fractions; 3 and 4 are enriched with organellar fractions and 5 with membrane fractions.
(TIF)

S6 Fig Alba-domain proteins do not localize to the endoplasmic reticulum or the mitochondrion. (A) Immunofluorescence images for assessing localization of HA-*Li*Alba3 and the endoplasmic reticulum chaperone BiP proteins in *L*. *infantum* exponentially grown promastigotes. *Li*Alba3^-/-^ parasites overexpressing pSP-alphaIRNEOalphaIR-HA-*Li*Alba3 were used for these studies. Immunolocalization was carried out as described in Materials and Methods using an anti-HA antibody (anti-mouse, green) and an anti-BiP antibody (anti-goat, red). (B) Immunofluorescence images of eYFP-*Li*Alba1 protein (green) in *L*. *infantum* exponentially grown promastigotes expressing pSP-NEOalphaIR-eYFP-*Li*Alba1. The mitochondrion was stained with MitoTracker (red).
(TIF)

S7 Fig The *Leishmania* Nop10 protein is localized to the nucleolus. Immunofluorescence images for mCh-*Li*Nop10 (red) localization in *L*. *infantum* promastigotes (Pro) and differentiating amastigotes (Diff) co-expressing pSP-NEOalphaIR-eYPF-*Li*Alba1 and pSP-HYGalphaIR-mCh-*Li*Nop10. The nucleus (N) and kinetoplastid DNA (K) were stained with DAPI (blue). Nu: nucleolus.
(TIF)

S8 Fig Alba-domain proteins are localized to the flagellum and the nucleolus only upon *Leishmania* amastigote differentiation. Subcellular localization of HA-*Li*Alba3 protein in *L*. *infantum* promastigotes (upper panel), during amastigote differentiation (8 h in MAA medium pH 5.8 at 37°C) (middle panel) and following switch of differentiating amastigotes into promastigote forms (SDM medium, 25°C, pH 7.0) (lower panel) was assessed by indirect immunofluorescence studies using an anti-HA antibody as described in Materials and Methods. DAPI staining (blue) allows detection of the nucleus (N) and kinetoplastid DNA (K).
(TIF)

S9 Fig Kinetics of mCherry-*Li*Alba3 flagellar accumulation during heat stress. *LiAlba3* ^-/-^ cells were episomally transfected with pSP-alphaIRNEOalphaIR-mCherry-*Li*Alba3 and exposed to heat stress for 8 h. Every 2 hours, aliquots were collected in parallel for i) microscopic analysis to assess the percentage of cells showing flagellar localization of *Li*Alba3 versus the total number of parasites visible by phase contrast; and ii) flow cytometry analysis to quantify the proportion of dead cells (sub-G1 phase) by staining the nucleus with propidium iodide.
(TIF)

S1 Table List of primers used in this study. (XLSX)

S2 Table Peptide and spectrum reports from LC-MS/MS analysis of *L*. *infantum* Alba1-HA and HA-Alba3 immunoprecipitation studies. (XLSX)

S3 Table Summary of the proteome analysis of *L*. *infantum* promastigote and differentiating amastigote flagellar fractions. (XLSX)

S4 Table Peptide and spectrum reports from LC-MS/MS analysis of the flagellum proteome of *L*. *infantum* promastigotes and differentiating amastigotes. (XLSX)
