# Supplementary figures and images for: Differential Subcellular Localization of Leishmania Alba-Domain Proteins throughout the Parasite Development
Source: PLoS One. 2015 Sep 3;10(9):e0137243. doi: 10.1371/journal.pone.0137243 (PMC4559404; doi:10.1371/journal.pone.0137243)

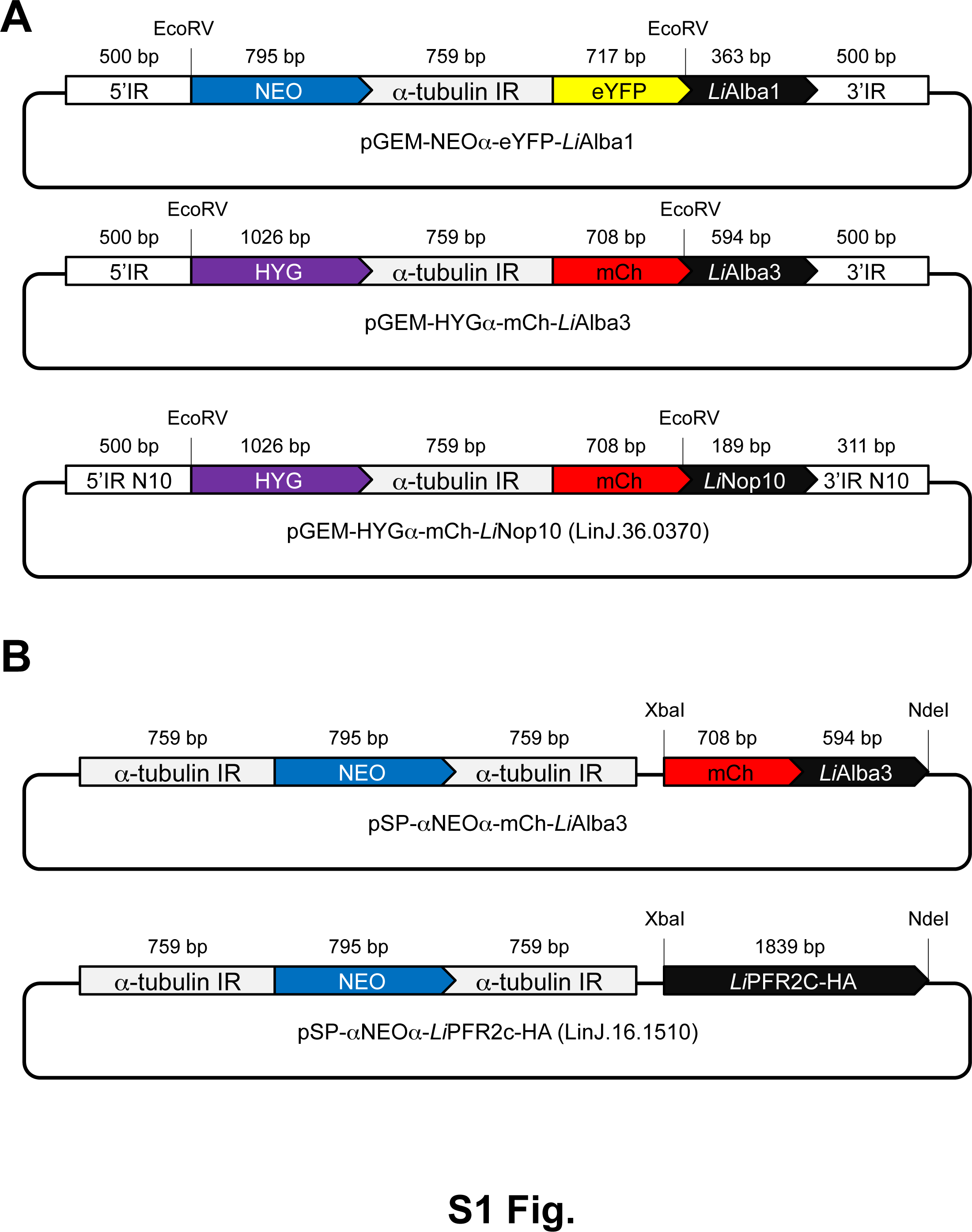

Supplement: S1 Fig — (A). The strategy we used for episomally expressing eYFP-LiAlba1, mCh-LiAlba3, and mCh-LiNop10 fluorescent proteins or for their genomic integration is the same for the three vectors. The fluorescent coding sequence (e.g. mCherry, and yellow fluorescent protein (eYFP)) was fused at the N-terminal part of LiAlba1, LiAlba3 or LiNop10 to keep the natural context of 3’UTR, hence allowing efficient regulation of the transcripts. The first cassette harboring a selectable marker gene (NEO or HYG), the alpha-tubulin intergenic region (alphaIR) for optimal processing, and the fluorescent protein without the stop codon was prepared by Phusion PCR. The NEO-alphaIR-eYFP and HYG-alphaIR-mCh cassettes were cloned into pGEM-T Easy vector for sequencing and further sub-cloning. The second step consisted in the amplification of a 500 bp fragment corresponding to the 5’-intergenic region of the target gene and the ORF together with the corresponding 3’-intergenic region (~500 bp). These two fragments were fused by PCR and an EcoRV site was added at the junction. (B) The mCh-LiAlba3 cassette was subcloned into pSP-alphaIRNEOalphaIR XbaI and NdeI sites to generate vector pSP-alphaIRNEOalphaIR-mCh-LiAlba3. PFR2C-HA was produced by PCR amplification and subcloned into pSP-alphaIRNEOalphaIR vector to generate pSP-alphaIRNEOalphaIR-PFR2C-HA. The primers used for PCR amplifications are listed in S1 Table. (TIF) [file pone.0137243.s001.tif]

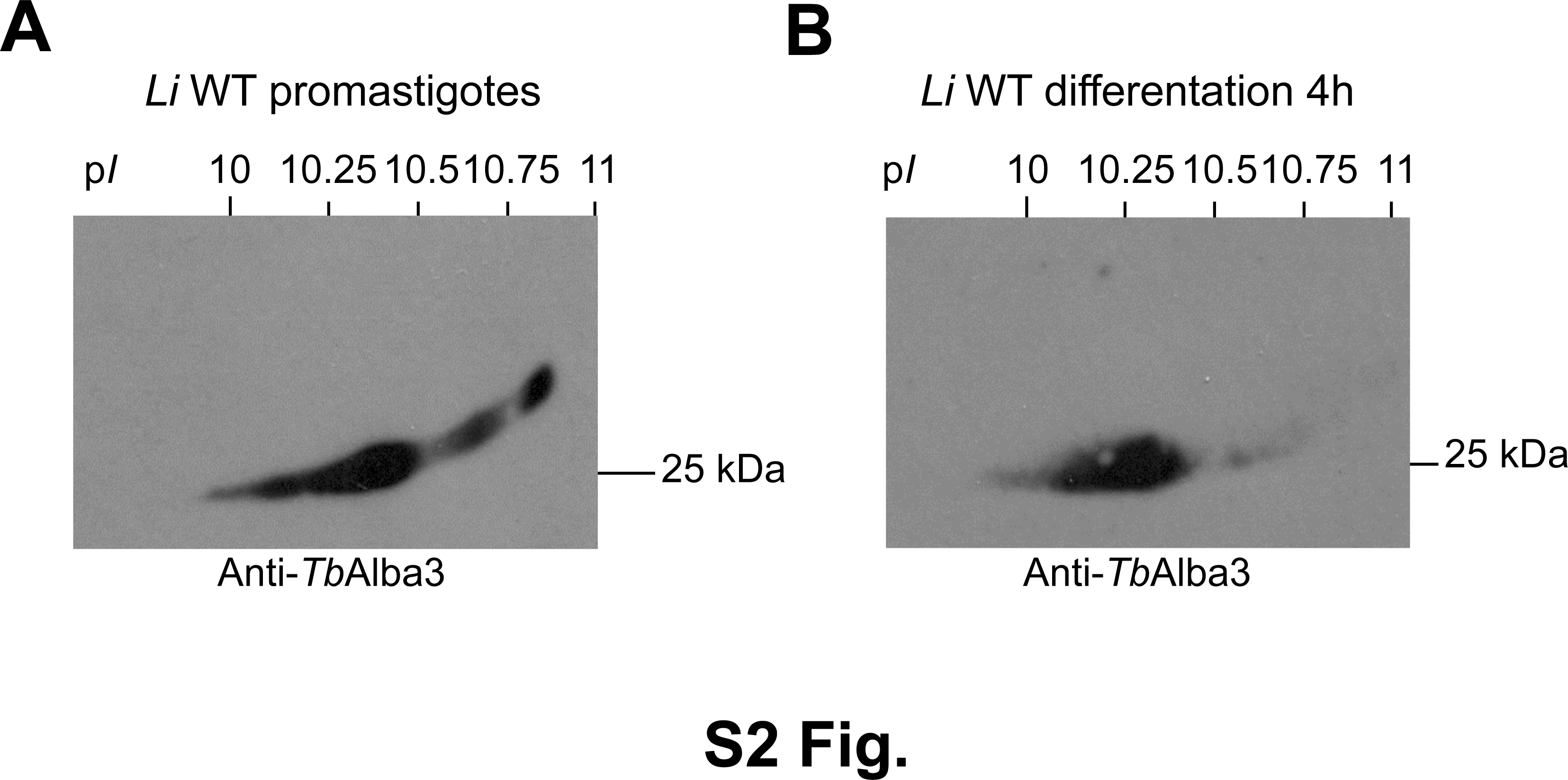

Supplement: S2 Fig — Total proteins from L. infantum promastigotes (A) or stationary promastigotes subjected to amastigote differentiation for 4 h (B) were loaded on a 2D gel (pH 6–11) and transferred on a nylon membrane for Western blotting using the T. brucei specific antibody recognizing the endogenous LiAlba3. The “smiling effect” is probably due to the migration and not to an increase in the molecular weight of LiAlba3 protein, as only a 25 kDa band was observed on 1D gels. pI between 10 and 11 is indicated on the top. (TIF) [file pone.0137243.s002.tif]

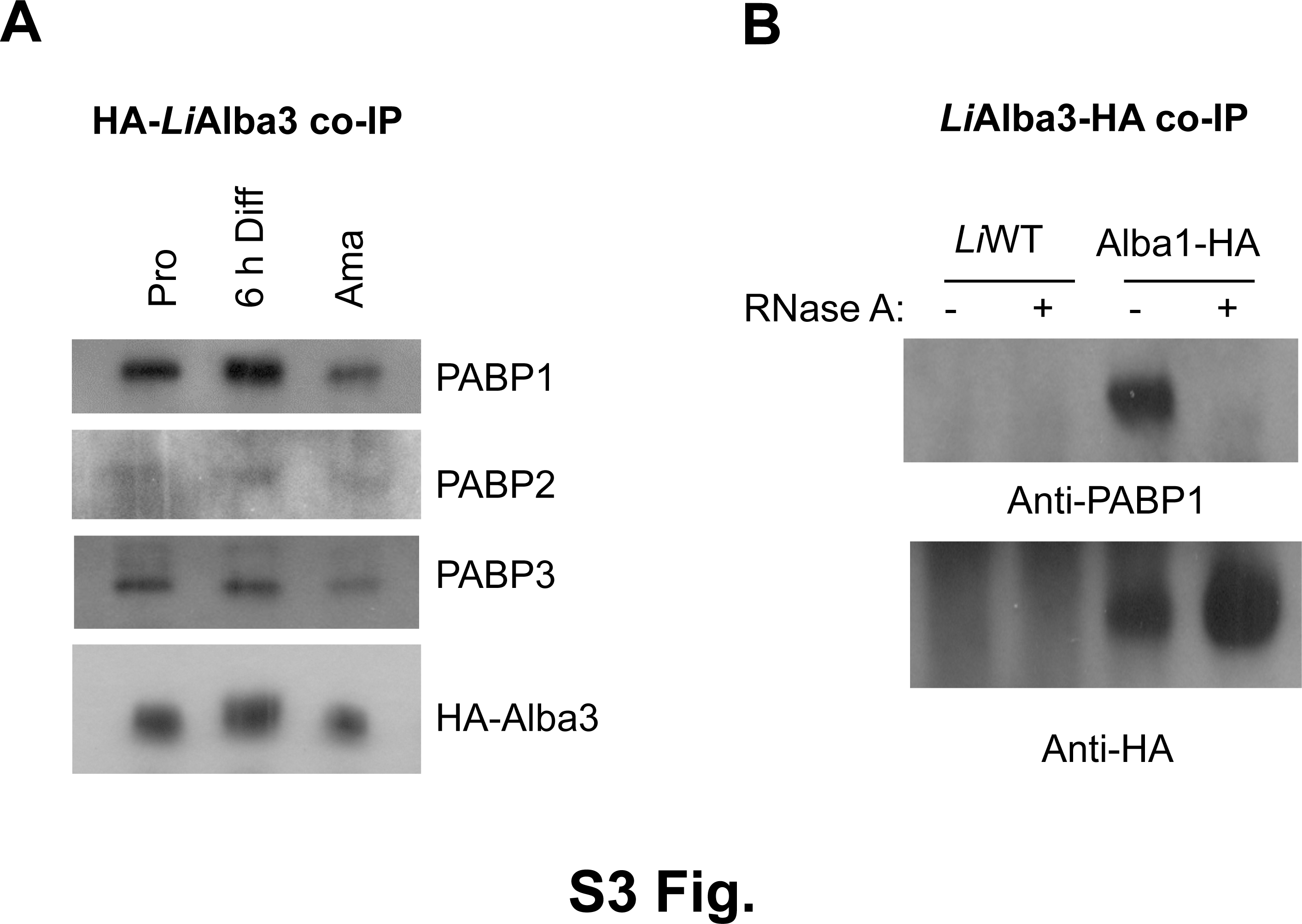

Supplement: S3 Fig — (A) Western blots of the HA-LiAlba3 pull-downs to confirm Alba-poly(A) binding protein (PABP) interactions under conditions of promastigote (Pro) or amastigote (Ama) growth or 6 h following amastigote differentiation (Diff) using antibodies made against the T. brucei PABP1-3 proteins that recognize the Leishmania othologs (antibodies kindly provided by Dr. Osvaldo de Melo Neto, Recife, Brazil; da Costa Lima TD et al., Eukaryot Cell 2010, 9(10):1484–94). (B) Western blot analysis of RNase A-treated samples prior to LiAlba1-HA co-immunoprecipitation using an antibody against PABP1 (upper panel) and an anti-HA antibody to detect LiAlba1-HA protein (lower panel). (TIF) [file pone.0137243.s003.tif]

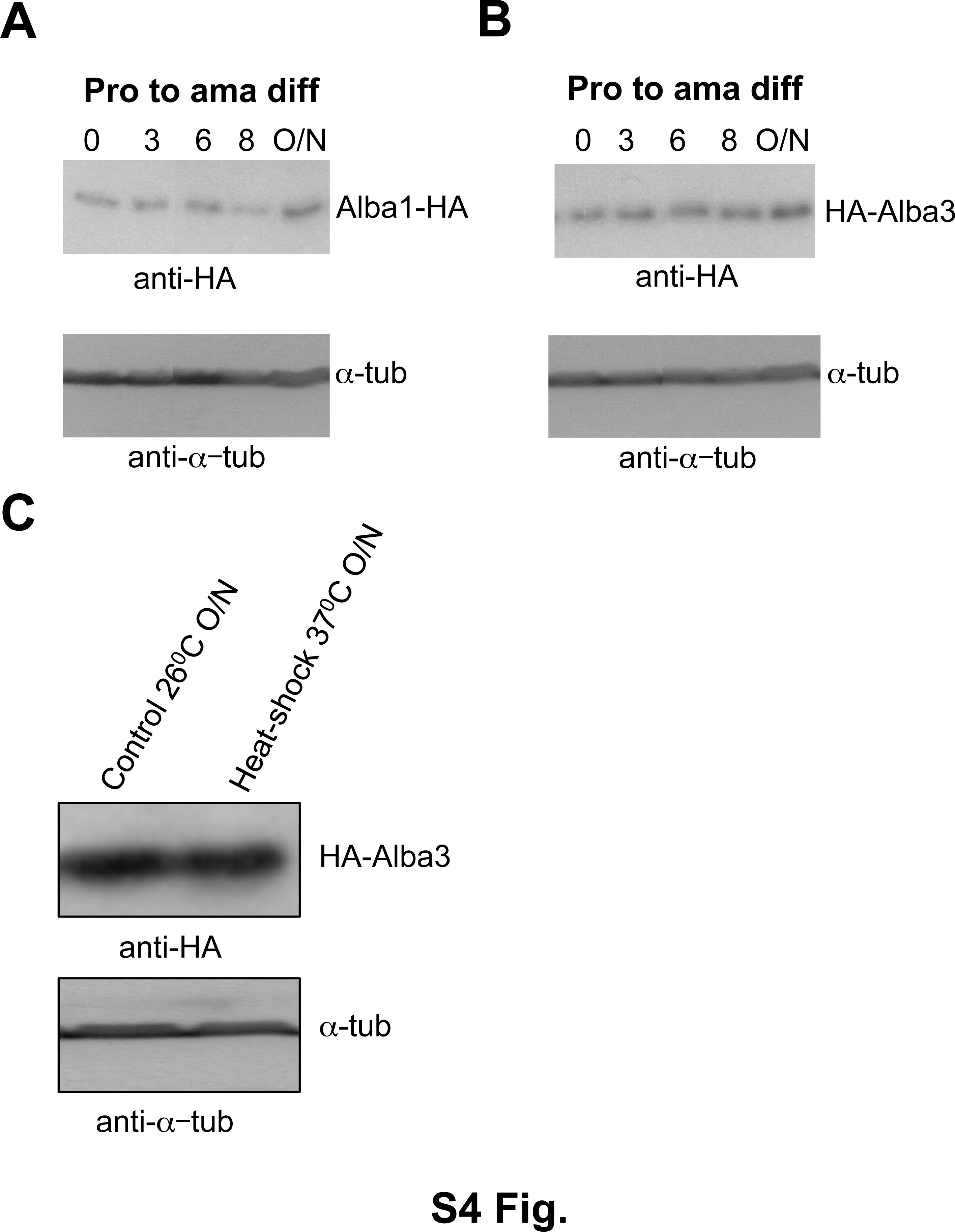

Supplement: S4 Fig — Western blot analysis using an anti-HA antibody to assess expression levels of the recombinant LiAlba1-HA (A) and HA-LiAlba3 (B) proteins during promastigote (pro) to amastigote (ama) differentiation (diff) or under heat stress (O/N) (C). An antibody against alpha-tubulin was used as loading control. (TIF) [file pone.0137243.s004.tif]

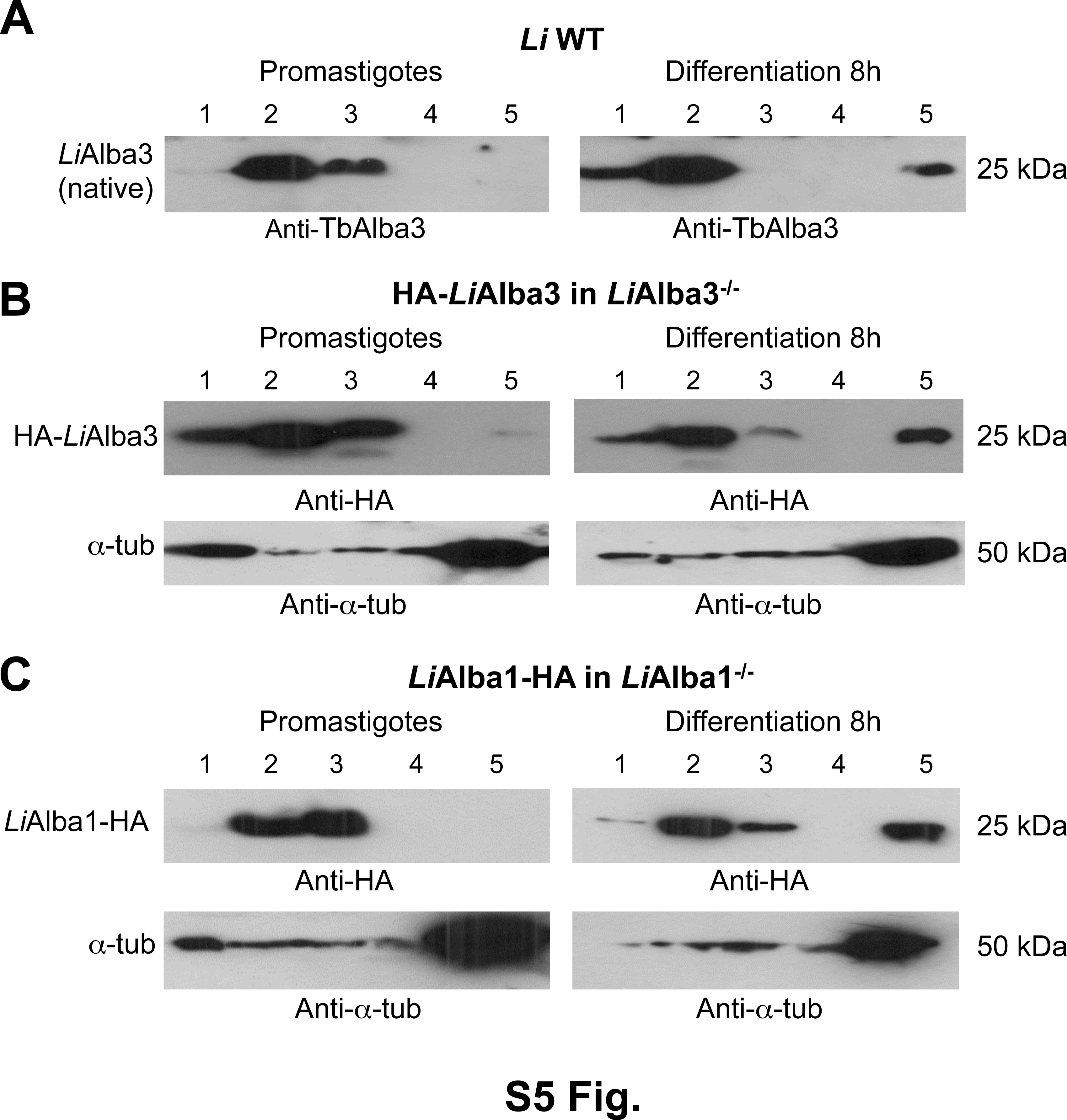

Supplement: S5 Fig — L. infantum wild type cells (WT) (A) and L. infantum add-back strains expressing HA-LiAlba3 (B) and LiAlba1-HA (C) into the LiAlba3-/- and LiAlba1-/- background, respectively, grown as promastigotes (exponential phase) or upon conditions of amastigote differentiation (MAA medium at pH 5.8 and 37°C for 8h) were used for these studies. Digitonin fractionation was done as previously described [63] and fractions from 1 to 5 were loaded on SDS-PAGE and transferred for Western blot analysis using specific antibodies against TbAlba3, HA, and alpha-tubulin. 1 and 2 correspond to cytosolic fractions; 3 and 4 are enriched with organellar fractions and 5 with membrane fractions. (TIF) [file pone.0137243.s005.tif]

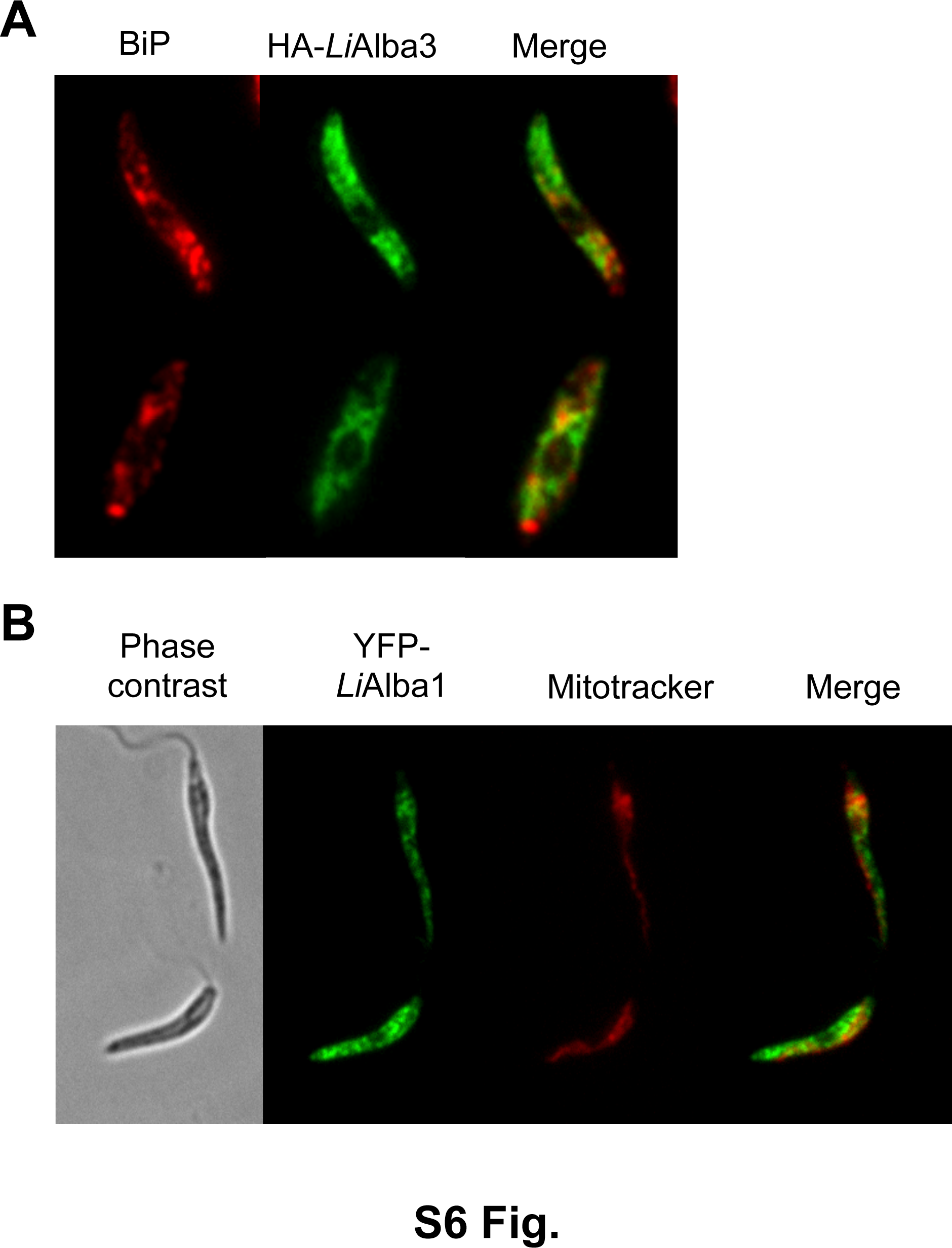

Supplement: S6 Fig — (A) Immunofluorescence images for assessing localization of HA-LiAlba3 and the endoplasmic reticulum chaperone BiP proteins in L. infantum exponentially grown promastigotes. LiAlba3-/- parasites overexpressing pSP-alphaIRNEOalphaIR-HA-LiAlba3 were used for these studies. Immunolocalization was carried out as described in Materials and Methods using an anti-HA antibody (anti-mouse, green) and an anti-BiP antibody (anti-goat, red). (B) Immunofluorescence images of eYFP-LiAlba1 protein (green) in L. infantum exponentially grown promastigotes expressing pSP-NEOalphaIR-eYFP-LiAlba1. The mitochondrion was stained with MitoTracker (red). (TIF) [file pone.0137243.s006.tif]

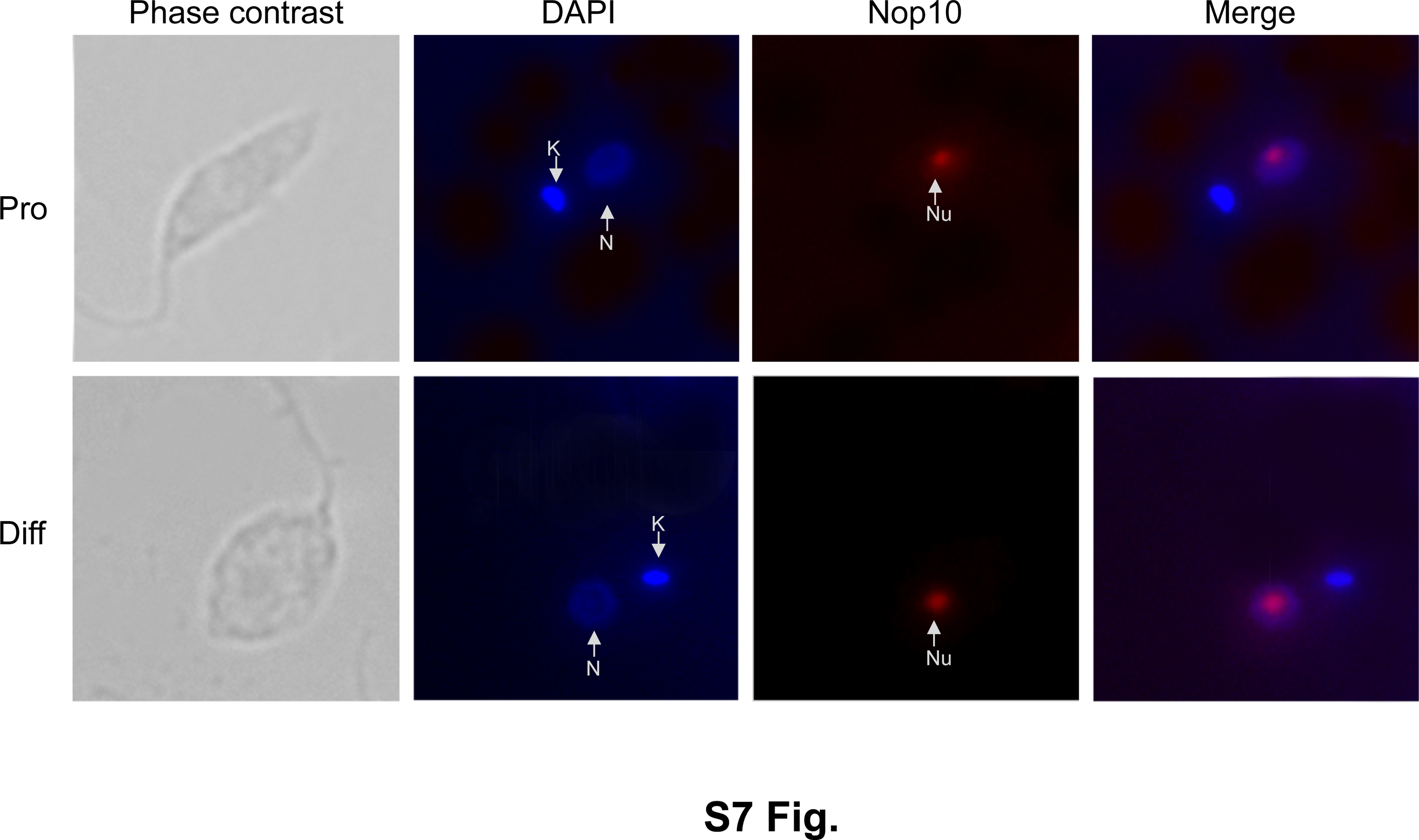

Supplement: S7 Fig — Immunofluorescence images for mCh-LiNop10 (red) localization in L. infantum promastigotes (Pro) and differentiating amastigotes (Diff) co-expressing pSP-NEOalphaIR-eYPF-LiAlba1 and pSP-HYGalphaIR-mCh-LiNop10. The nucleus (N) and kinetoplastid DNA (K) were stained with DAPI (blue). Nu: nucleolus. (TIF) [file pone.0137243.s007.tif]

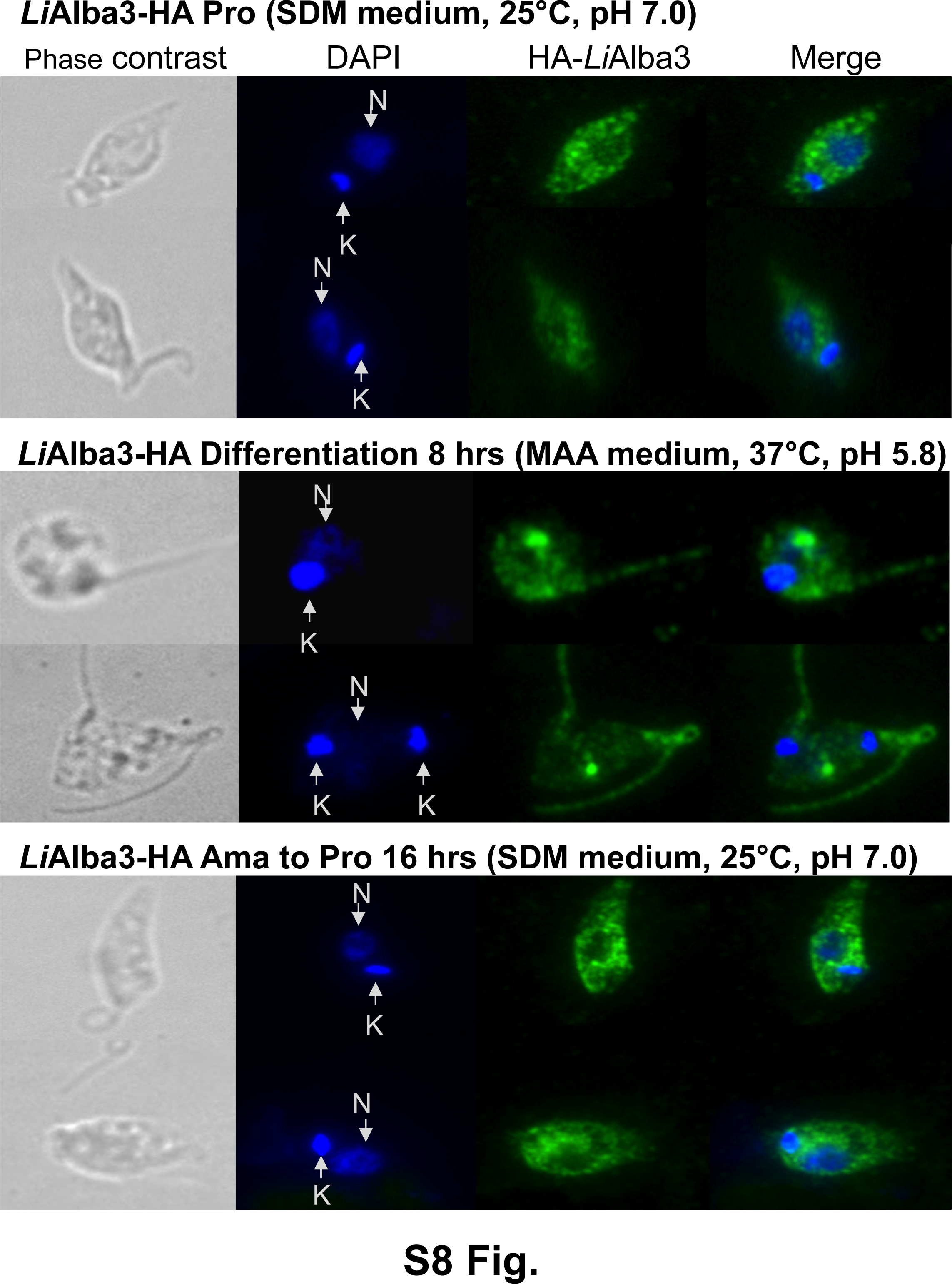

Supplement: S8 Fig — Subcellular localization of HA-LiAlba3 protein in L. infantum promastigotes (upper panel), during amastigote differentiation (8 h in MAA medium pH 5.8 at 37°C) (middle panel) and following switch of differentiating amastigotes into promastigote forms (SDM medium, 25°C, pH 7.0) (lower panel) was assessed by indirect immunofluorescence studies using an anti-HA antibody as described in Materials and Methods. DAPI staining (blue) allows detection of the nucleus (N) and kinetoplastid DNA (K). (TIF) [file pone.0137243.s008.tif]

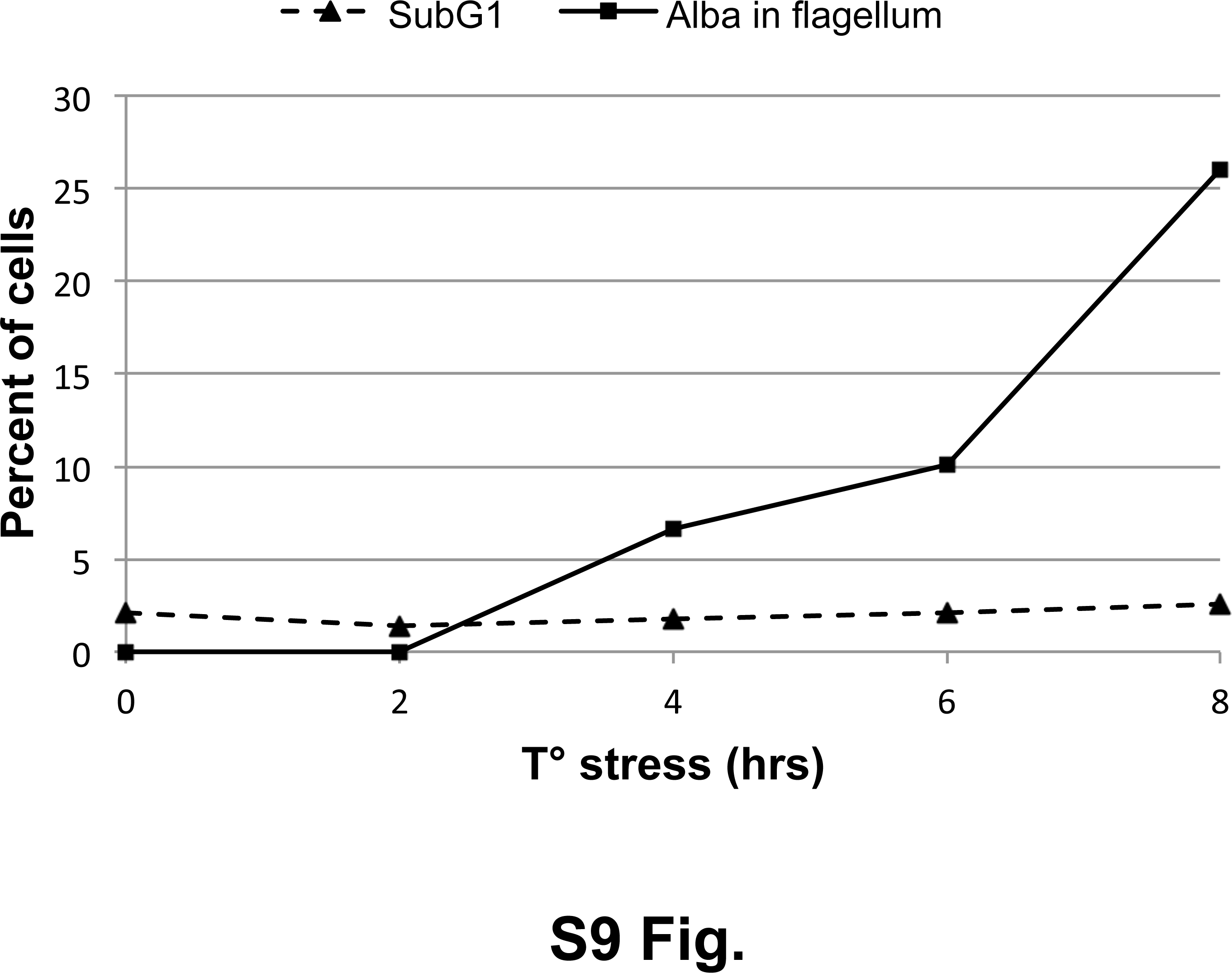

Supplement: S9 Fig — LiAlba3 -/- cells were episomally transfected with pSP-alphaIRNEOalphaIR-mCherry-LiAlba3 and exposed to heat stress for 8 h. Every 2 hours, aliquots were collected in parallel for i) microscopic analysis to assess the percentage of cells showing flagellar localization of LiAlba3 versus the total number of parasites visible by phase contrast; and ii) flow cytometry analysis to quantify the proportion of dead cells (sub-G1 phase) by staining the nucleus with propidium iodide. (TIF) [file pone.0137243.s009.tif]
